# Fungal Community Development in Decomposing Fine Deadwood Is Largely Affected by Microclimate

**DOI:** 10.3389/fmicb.2022.835274

**Published:** 2022-04-13

**Authors:** Vendula Brabcová, Vojtěch Tláskal, Clémentine Lepinay, Petra Zrůstová, Ivana Eichlerová, Martina Štursová, Jörg Müller, Roland Brandl, Claus Bässler, Petr Baldrian

**Affiliations:** ^1^Laboratory of Environmental Microbiology, Institute of Microbiology of the Czech Academy of Sciences, Prague, Czechia; ^2^Department of Animal Ecology and Tropical Biology, University of Würzburg, Würzburg, Germany; ^3^Bavarian Forest National Park, Grafenau, Germany; ^4^Animal Ecology, Department of Ecology, Faculty of Biology, Philipps-Universität Marburg, Marburg, Germany; ^5^Department of Conservation Biology, Faculty of Biological Sciences, Institute for Ecology, Evolution and Diversity, Goethe University Frankfurt, Frankfurt, Germany

**Keywords:** decomposition, deadwood, fungal community, succession, canopy cover, microclimate, temperate forest, ecology

## Abstract

Fine woody debris (FWD) represents the majority of the deadwood stock in managed forests and serves as an important biodiversity hotspot and refuge for many organisms, including deadwood fungi. Wood decomposition in forests, representing an important input of nutrients into forest soils, is mainly driven by fungal communities that undergo continuous changes during deadwood decomposition. However, while the assembly processes of fungal communities in long-lasting coarse woody debris have been repeatedly explored, similar information for the more ephemeral habitat of fine deadwood is missing. Here, we followed the fate of FWD of *Fagus sylvatica* and *Abies alba* in a Central European forest to describe the assembly and diversity patterns of fungal communities over 6 years. Importantly, the effect of microclimate on deadwood properties and fungal communities was addressed by comparing FWD decomposition in closed forests and under open canopies because the large surface-to-volume ratio of FWD makes it highly sensitive to temperature and moisture fluctuations. Indeed, fungal biomass increases and pH decreases were significantly higher in FWD under closed canopy in the initial stages of decomposition indicating higher fungal activity and hence decay processes. The assembly patterns of the fungal community were strongly affected by both tree species and microclimatic conditions. The communities in the open/closed canopies and in each tree species were different throughout the whole succession with only limited convergence in time in terms of both species and ecological guild composition. Decomposition under the open canopy was characterized by high sample-to-sample variability, showing the diversification of fungal resources. Tree species-specific fungi were detected among the abundant species mostly during the initial decomposition, whereas fungi associated with certain canopy cover treatments were present evenly during decomposition. The species diversity of forest stands and the variability in microclimatic conditions both promote the diversity of fine woody debris fungi in a forest.

## Introduction

Temperate forests represent a significant global sink of carbon (C) (Harris et al., 2021). Part of the C removed from the atmosphere is incorporated into tissues of the trees—wood, leaves, and roots. The amount of C stored in deadwood varies considerably among natural temperate forests of Central Europe, reaching up to 550 m^3^ ha^–1^ (Christensen et al., 2005; Král et al., 2010; Seibold et al., 2021). Beech (*Fagus sylvatica*) forests with a variable proportion of fir (*Abies alba*) are among the most common forests in Central Europe (Krah et al., 2018), where they represent the natural vegetation of submontane to montane forests (Bolte et al., 2007). Fine woody debris (FWD, deadwood of diameter < 10 cm) represents only a small portion of deadwood stocks in natural forests where snags and coarse wood of fallen trees are present (Baldrian, 2017; Ricker et al., 2019). Although the amount of fine deadwood in temperate forests estimated typically at 2–9 m^3^ ha^–1^ (Nordén et al., 2004; Domke et al., 2016) appears moderate, it represents the dominant stock of deadwood in managed forests where tree stems are harvested. Whereas coarse woody debris (CWD) decomposes slowly and persists over multiple decades (Přívětivý et al., 2018), FWD shows rapid turnover due to fast decomposition (Müller-Using and Bartsch, 2009). Moreover, fragmentation of the FWD resource creates a high variety of habitats and a high number of habitat patches, leading to a high diversity of fungi (Heilmann-Clausen and Christensen, 2004). FWD decomposition might be affected by multiple factors, including tree species, size, and climatic conditions at a site (Berbeco et al., 2012; Ricker et al., 2016), with reported rates of mass loss of 0.17–0.25 year^–1^ (Fasth et al., 2011; Ostrogović et al., 2015).

In forest ecosystems, deadwood decomposition represents one of the paths of nutrient input into soils (Peršoh and Borken, 2017; Šamonil et al., 2020). These nutrients originate in the plant biomass (Šamonil et al., 2020) or, in the case of nitrogen, are fixed from the atmosphere by deadwood bacteria (Rinne et al., 2017; Tláskal et al., 2021) and enter topsoil after full deadwood decomposition. Deadwood is also one of the most important factors contributing to the maintenance of biodiversity in forests (Stokland et al., 2012; Seibold et al., 2015). Namely, it is a habitat and a nutrient source for a wide range of organisms, including microorganisms, fungi and bacteria (Johnston et al., 2016; Větrovský et al., 2020; Tláskal and Baldrian, 2021). Due to filamentous growth, possession of extracellular enzymes and the ability to acidify their substrate, fungi are efficient in the colonization of large patches of wood and its utilization, despite the recalcitrance, fluctuating moisture content and low nitrogen content of this substrate.

The study of fungi on decomposing wood in the past typically targeted CWD (Rajala et al., 2012; Arnstadt et al., 2016; Baldrian et al., 2016), except for the pioneering studies analyzing microbes on decomposing *Salix caprea* twigs (Angst et al., 2018) or substrate preferences of fungi on deadwood of various sizes (Juutilainen et al., 2017). Fungal communities of FWD were typically studied by fruitbody surveys, although the fruitbody approach has several important limitations, such as limited sporocarp production on small woody resources (Ovaskainen et al., 2013). FWD was reported to support higher fungal diversity than the CWD of a corresponding volume (Heilmann-Clausen and Christensen, 2004; Nordén et al., 2004; Bässler et al., 2010). In a comparative survey, 75% of Ascomycota and 30% of Basidiomycota species were found exclusively on FWD (Nordén et al., 2004), and the majority of wood-inhabiting basidiomycetes were found on branches with diameters < 5 cm (Küffer and Senn-Irlet, 2005). Conventional forest management practices remove CWD and change microclimate conditions due to manipulation of the canopy (Bässler et al., 2014). Under such conditions, FWD at clearcuts and in forest stands can serve as an important refuge for wood fungi (Nordén et al., 2004; Küffer and Senn-Irlet, 2005; Juutilainen et al., 2014).

Multiple factors affect fungal communities on deadwood, including tree species, deadwood size or wood decay stage (Juutilainen et al., 2014; Atrena et al., 2020), but among stand-level variables, canopy gaps are also important (Krah et al., 2018; Atrena et al., 2020). The effect of tree species is broadly attributed to the species-specific differences in deadwood properties, namely, in density, content of nitrogen or the amounts and origins of phenolics and other extractives such as resins. These factors lead to different decomposition rates, organismic diversity and successive changes in the compositions and activities of wood decomposing fungal communities (Kahl et al., 2017; Angst et al., 2019). pH and the carbon/nitrogen ratio are strong predictors of the fungal community composition affecting the abundance of dominant fungal taxa (Lepinay et al., 2021a), and their changes goes hand-in-hand with successional changes in fungal communities. Fungi with different morphological traits inhabit deadwood of different qualities, leading to the specialization of the fungal communities and organismic diversity of fungal colonizers (Purhonen et al., 2020). The canopy openness of forests is closely related to their temperature buffering capacity (Frey et al., 2016; De Frenne et al., 2019). Forest gaps are characterized by an increased respiration rate (Forrester et al., 2012), elevated summer temperatures, higher solar radiation, and changed soil water content (Scharenbroch and Bockheim, 2007). Considering that climatic factors are the primary drivers of fungal distribution (Větrovský et al., 2019), FWD seems to be especially sensitive to actual microclimatic conditions due to its high surface-to-volume ratio (Bässler et al., 2010). However, the extent to which microclimate affects fungi in decomposing FWD is not known.

In this study, we set up an experiment where the fates of the FWD of beech (*Fagus sylvatica*) and fir (*Abies alba*), the two main tree species of the temperate forests of central Europe at elevations of 750–900 m a. s. l., were followed for 6 years under open and closed canopies. The aim was to determine the factors affecting the assembly of fungal communities during decomposition and assess the size and relative importance of the effects of microclimate and the host tree. Experimental plots with sunny gaps surrogated the changed microclimatic conditions caused by natural disturbances such as windstorms, insect outbreaks, or forest management. The frequency of these events has increased in the temperate zone, and they have more pronounced effects in managed forests than in unmanaged forests (Sommerfeld et al., 2018). The length of the experiment covered an extended part of FWD decomposition and lasted until 2017, when mass loss and fragmentation made it impossible to follow FWD at certain locations.

We hypothesized that beech and fir FWDs are inhabited by a substantial share of tree species-specific fungal taxa and tree species are therefore the key factor affecting fungal community. Host tree species were reported as an important driver of fungal community development on coarse deadwood (Nordén et al., 2004; Baber et al., 2016; Krah et al., 2018; Atrena et al., 2020), although not equally strong in all cases (Baldrian et al., 2016). We expected a high share of *r*-selected species, namely, molds and yeasts (Mašínová et al., 2018; Algora Gallardo et al., 2021), in the earlier stages of fine deadwood decomposition, similar to the decomposition of leaf and plant litter (Voříšková and Baldrian, 2013). We further hypothesized that higher canopy openness results in decreased decomposition due to harsher conditions and selection of specific taxa adapted to the actual microclimatic conditions. To evaluate the importance of the FWD as an ecosystem component, we also quantified the amount of FWD present in the temperate forest of the study area representing the typical managed forest in the mountains of Central Europe.

## Materials and Methods

### Study Area and Experimental Design

The experimental sites were located in the management zone of the Bavarian Forest NP in Germany (48.9° N, 13.3°E). The management zone covers an area of 6,000 ha that surrounds the 18,000 ha core zone of the national park. The area is characterized by montane mixed forest consisting of European beech (*Fagus sylvatica* L.), Silver fir (*Abies alba* Mill.) and Norway spruce [*Picea abies* (L.) H. Karst] (Bässler et al., 2010). The sampling design was a part of the broader experimental design described in Krah et al. (2018). In autumn 2011, freshly cut branches of fir and beech were deposited on 64 plots and arranged in a random block design with four spatially independent blocks. The branches (fine woody debris, FWD) had diameters of 3.2 ± 1.3 cm and lengths of 2.7 ± 0.9 m. These branches were taken from trees of the same age that were harvested from the same forest stand. The origin of the branches was identical and branches were randomly distributed across study sites to mitigate the potential effect of communities of fungal endophytes inhabiting individual branches on fungal community development. Each block contained randomly located sets of plots with either fir or beech branches or both. The mixture of fir and beech deadwood represented the factor of forest stand tree diversity. Within each block, two plots per treatment (fir, beech, or mixed) were set under open or closed canopies (Supplementary Figure 1). Canopy openness was used as a surrogate for stand microclimates (Müller et al., 2015; Seibold et al., 2015; Krah et al., 2018; De Frenne et al., 2019). The sunny open canopy plots were the result of clearings where an area of 0.1 ha was freed from living and dead trees. To avoid shading by a dense grass layer surrounding the deadwood on sunny plots, each plot was mowed once a year during the growing season as described previously (Seibold et al., 2016a,b). The daily peak temperatures of the deadwood surfaces in summer were measured in the sunny and shady plots. The mean values were much higher in sunny plots (∼30°C) than in the closed canopies (∼15°C) (Müller et al., 2020). All experimental plots were sampled annually in October from 2012 to 2017.

### Fine Woody Debris Census

To quantify the local stock of FWD within the unmanaged forest, FWD was collected from the litter surface and organic horizon of soil in thirty-six 2 × 2 m squares located close to the experimental sites. All FWD inside the square was collected, as well as the FWD intersecting the eastern and southern borders of the square, while the FWD intersecting the western and northern borders was disregarded. The FWD was classified into three size categories (diameters of the thicker end of the FWD of 0.5–1.5, 1.6–5.0, and 5.1–10.0 cm). Per-square counts and masses of the FWD were recorded. FWD from each size category was subsampled and weighed before and after air-drying to assess dry mass content. The per-square FWD mass was estimated by multiplying the wet mass per plot by the relative dry mass content of each size category. Collected FWD was not used in set experiment as it included FWD of different decay length.

### Sampling, Sample Processing, and Analysis

One composite FWD sample was obtained from each selected branch. It was obtained from two vertical drillings of the branch in its center using an electric drill equipped with a 10 mm diameter auger across the entire diameter of the branch. The drilling points were placed evenly along the branch, avoiding the close proximity of the ends of the branch. The auger was sterilized between drillings, and the dust from all drilling points was collected in sterile plastic bags and frozen within a few hours after drilling. In total, 2 beech or 2 fir samples were taken from each block from plots containing only beech or fir, and 2 beech and 2 fir samples were taken from plots containing mixed deadwood, resulting in 4 beech and 4 fir composite samples per canopy type. Thus 64 samples in total were taken annually (Supplementary Figure 1), resulting in a total of 384 samples in the study.

The drilled materials were weighed in the laboratory and freeze-dried to estimate the deadwood dry mass. Next, it was milled using an Ultra Centrifugal Mill ZM 200 (Retsch, Germany), and the resulting fine powder was used for the subsequent analyses. Dry mass content was based on the loss of mass during freeze-drying, and pH was measured after mixing with distilled water (1:10 w:vol). The wood C and N contents were measured using an elemental analyzer in an external laboratory of the Institute of Botany of the Czech Academy of Sciences, Průhonice, Czech Republic, as described previously (Větrovský and Baldrian, 2015). C was measured using sulfochromic oxidation, and the nitrogen content was estimated by sulfuric acid mineralization with the addition of selenium and sodium sulfate and conversion to ammonium ions, which were measured by a segmented flow analyzer (SFA), Skalar. To quantify fungal biomass, total ergosterol was extracted using 10% KOH in methanol and analyzed by HPLC (Šnajdr et al., 2008).

### Extraction and Analysis of Environmental DNA

Total genomic DNA was extracted from 200 mg of freeze-dried material using the NucleoSpin Soil Kit (Macherey-Nagel, Germany) according to the manufacturer’s instructions (Baldrian et al., 2016). Briefly, cells were lysed using SL1 lysis buffer. Enhancer SX was added prior to lysis. The samples were homogenized using FastPrep-24 (MP Biomedicals, Santa Anna, United States) at 5 m s^–1^ for 2 × 30 s. In the last step, DNA was eluted from the columns using 50 μl of deionized water. One extraction per sample was performed.

For the microbial community analysis, PCR amplification of the fungal ITS2 region was performed using barcoded gITS7 and ITS4 primers (Ihrmark et al., 2012) in triplicate PCRs per sample as described previously (Baldrian et al., 2016). PCRs contained 2.5 μl of 10 × buffer for DyNAzyme DNA Polymerase, 0.75 μl of BSA (20 mg ml^–1^), 1 μl of each primer (0.01 mM), 0.5 μl of PCR Nucleotide Mix (10 mM each), 0.75 μl polymerase (2 U μl^–1^ DyNAZyme II DNA polymerase 1: 24 Pfu DNA polymerase) and 1 μl of template DNA. Cycling conditions were 94°C for 5 min, 35 cycles of 94°C for 1 min, 62°C for 1 min, and 72°C for 1 min, and a final extension at 72°C for 10 min. PCR triplicate reaction products were pooled and purified, and amplicon libraries prepared with the TruSeq DNA PCR-Free Kit (Illumina) were sequenced in house on the Illumina MiSeq (2 × 250-base reads).

The amplicon sequencing data were processed using the pipeline SEED 2.1.1 (Větrovský et al., 2018). Briefly, paired-end reads were merged using fastq-join (Aronesty, 2013). The ITS2 region was extracted using ITS Extractor 1.0.11 (Bengtsson-Palme et al., 2013) before processing. Chimeric sequences were detected using Usearch 11.0.667 (Edgar, 2010) and deleted, and sequences were clustered using UPARSE implemented within Usearch (Edgar, 2013) at a 97% similarity level. The most abundant sequences were selected from each cluster, and the closest hits at the species level were identified using BLASTn against UNITE (Nilsson et al., 2019). Where the best hit showed lower similarity than 97 with 95% coverage, the best genus-level hit was identified. Species-level analyses were performed on a dataset where OTUs belonging to the same species were combined and all other OTUs were combined into the genus of the best hit and designated “sp.” Sequences identified as *Fagus* sp. or *Abies* sp. were discarded similarly as well as non-fungal sequences. Sequencing data have been deposited in the SRA database under BioProject accession number PRJNA671809.

To assign putative ecological functions to the fungal OTUs, the fungal genera of the best hit were classified into ecological categories (e.g., white rot, brown rot, saprotroph, plant pathogen, ectomycorrhiza) based on (Põlme et al., 2020). Fungal OTUs not assigned to a genus with known ecophysiology and those assigned to genera with unclear ecology remained unclassified.

### Data Processing and Statistics

*Succession time* was defined as the average position of a taxon in succession considering its relative abundance over time, and *the duration of occurrence* was defined as the time span covering 90% of the taxon relative abundance as defined and calculated previously (Štursová et al., 2020). Tree or canopy specificity was defined as the strength of association of the taxon with one particular tree or canopy type and calculated as the sum of abundances in beech deadwood (closed canopy deadwood) divided by the sum of abundances in all samples. A value of 1 corresponds to a taxon exclusively found on beech deadwood. A value of 0.5 assigned to a taxon indicates that it is equally abundant on beech and fir deadwood. A value of 0 assigned to a taxon indicates that it is exclusively found on fir deadwood. For canopy openness, a value of 1 corresponds to a taxon exclusively found under the closed canopy and 0 corresponds to a taxon exclusively found under the open canopy. Taxa with tree specificities between 1.0 and 0.95 were considered beech-specific (or closed canopy-specific, respectively), and those with specificities between 0.05 and 0.00 were considered fir-specific (or open canopy-specific, respectively).

Statistical analyses were performed in PAST 4.03^1^ and R (R Core Team, 2020). Two-dimensional non-metric multidimensional scaling (NMDS) ordination analysis on Euclidean distances was used to address the dissimilarity of the fungal community compositions based on Hellinger-transformed relative abundances. NMDS was performed in R with the package *vegan*, function metaMDS (Oksanen et al., 2020; R Core Team, 2020). Variables were fitted to the ordination diagram as vectors with 999 permutations and included pH, as well as the C and N and ergosterol contents. Diversity estimates (Shannon–Wiener index, OTU richness, Chao 1, and evenness) were calculated for a dataset containing the relative abundance of 2,000 randomly selected sequences from each sample in SEED 2.1.1 (Větrovský et al., 2018). Differences in the environmental variables (pH, C, N, C/N, ergosterol, and water contents) were tested using a linear mixed model (LMM, function lmer) with log-transformed data. For the LMM, the effect of explanatory variables, i.e., tree species, canopy openness and their interaction, over decomposition time were tested by considering time and plot identifiers (the same plots were repeatedly measured over time) as random effects. Differences between the explanatory variables were also tested for each time separately using two-way ANOVA or the non-parametric Kruskal–Wallis test and Tukey–Kramer HSD test on log-transformed data. Mantel tests with 99,999 permutations were used to examine the correlations between data matrices, and Euclidean distances were used for all variables except community data. One-way or two-way PERMANOVA tests with 9,999 permutations were used to examine the effects of treatments on fungal communities. Spearman rank correlations were used as a measure of the relationships between variables. Variation partitioning analyses on Hellinger-transformed OTU abundances were performed to identify the parts of the variance explained by tree species and canopy openness for the whole dataset and with length of decomposition, canopy openness and wood chemistry (i.e., N, C, C/N and pH) for each tree species independently. Data were rarefied to 2,000 sequences. The “varpart” function from the “vegan” R package was used (Oksanen et al., 2020). The importance values of the obtained variances were determined with Monte Carlo permutation tests. In all cases, differences at *P* < 0.05 were considered statistically significant.

## Results

### Abundance and Moisture of Fine Woody Debris

The abundance of fine woody debris at the tested small-scale plots of 2 × 2 m exhibited high spatial variability, and their masses ranged from 1.4 to 11.50 t ha^–1^ dry mass (90,000–230,000 pieces per ha). On average, 5.3 ± 2.8 t ha^–1^ fine woody debris was recorded. This was composed of 1.0 ± 0.5 t ha^–1^ (19%) of twigs with diameters of < 1.5 cm, 1.9 ± 1.3 t ha^–1^ (35%) of branches with diameters between 1.6 and 5 cm, and 2.4 ± 2.7 t ha^–1^ (46%) of branches with diameters between 5.1 and 10 cm. The mean moisture content of fine woody debris was 26.2 ± 1.8% and it was almost equivalent in all size classes (Supplementary Figure 2).

### Properties of Fine Woody Debris During Six Years of Decomposition

**FIGURE 1:**
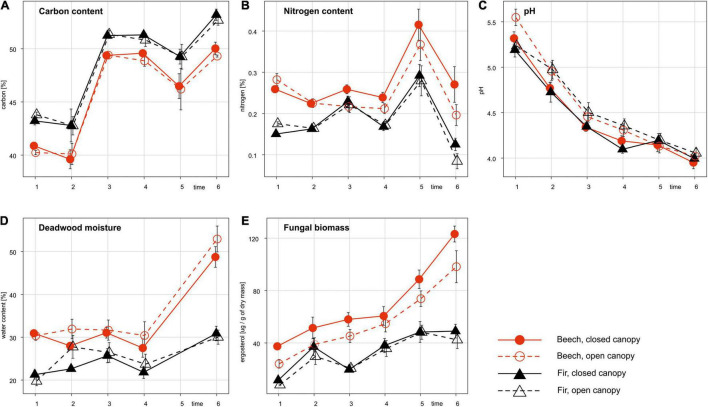
Fungal biomass **(E)** and changes in physicochemical compositions **(A–D)** of fine beech and fir deadwoods during decomposition in a natural temperate forest. Data represent the mean ± SE of 16 samples per treatment and timepoint (years).

The chemical and nutritional compositions of FWD differed considerably between tree species. Beech and fir were characterized by different C:N ratios in the first year of decomposition [beech C:N = 154 ± 5, fir C:N = 270 ± 7, *F*_(1, 57)_ = 231.66, *P* < 0.001] due to the lower content of N in fir deadwood [0.27 ± 0.01% in beech, 0.16 ± 0.00% in fir, *F*_(1, 57)_ = 145.70, *P* < 0.001]. Overall, the carbon contents increased during decomposition (LMM: χ^2^ = 5.89, *P* = 0.015), while the N contents fluctuated over time (LMM: χ^2^ = 0.04, *P* = 0.834). Beech deadwood had, on average, higher moisture content over the whole experiment, and increased in time. Canopy cover had no effect on deadwood moisture content at the time of sampling in October (Figure 1). The pH of both tree species decreased steadily from 5.3 to 4.0 during decomposition (LMM: χ^2^ = 14.56, *P* < 0.001). Beech deadwood differed in pH from fir deadwood only in the first year [*F*_(1, 57)_ = 9.41, *P* = 0.003]. The pH was significantly lower under the closed canopy in years 2 and 4 of decomposition [*F*_(1, 58)_ = 4.97, *P* = 0.030 and *F*_(1, 58)_ = 8.49, *P* = 0.005, respectively]. The diversity of the deadwood in the plots (single tree or mixed species) had no effect on the deadwood pH, deadwood moisture, or nutrient contents [*F*_(1, 58)_ < 0.56, *P* > 0.457 for all estimates].

### Fungal Biomass and Community Compositions in the Different Types of Fine Woody Debris

The fungal biomass content quantified as ergosterol was significantly higher in beech than in fir deadwood throughout the whole experiment (LMM: χ^2^ = 16.61, *P* < 0.001). On average it was twice as high (Figure 1). While the ergosterol content in beech deadwood increased steadily during the whole experiment from 31 μg g^–1^ in year 1 to 110 μg g^–1^ in year 6, in fir deadwood, it increased only within the first 5 years from 10 to 48 μg g^–1^ and then remained stable. The canopy cover effect was important after 1 year of decomposition, with higher fungal biomass under the closed canopy [*F*_(1, 58)_ = 24.13, *P* < 0.001; Figure 1]; higher diversity of deadwood origin on the plot had no effect on fungal biomass [*F*_(1, 58)_ = 2.51, *P* = 0.115 for fir and *F*_(1, 58)_ < 0.01, *P* = 0.949 for beech].

The fungal diversity estimated as species richness or the Chao-1 index was significantly higher in fir FWD in canopy gaps than under closed canopy gaps (*P* < 0.0001). The species richness overall was highest in beech FWD after 5 years of decomposition, and in fir FWD after 4 years of decomposition while it decreased later (Supplementary Figure 3). Among the potential drivers of fungal community composition, one-way PERMANOVA and variation partitioning analyses showed that tree species, canopy cover and time were all significant (*P* < 0.0001, one-way PERMANOVA). Most of the variation was explained by tree species (8.3%) and canopy cover (8.0%) (Supplementary Figure 4). Within the separate beech and fir deadwoods, the canopy type and time as well as their interaction had significant effects on fungal communities (*P* < 0.0001, two-way PERMANOVA). Time had clearly a lower impact on fungal community composition than tree species and canopy type (tree *F* = 24.3, canopy *F* = 26.6, time *F* = 5.2, all *P* < 0.0001). Canopy cover explained 11.8 and 12.7% of the detected variability in beech and fir FWD, respectively (Supplementary Figures 4B,C).

**FIGURE 2:**
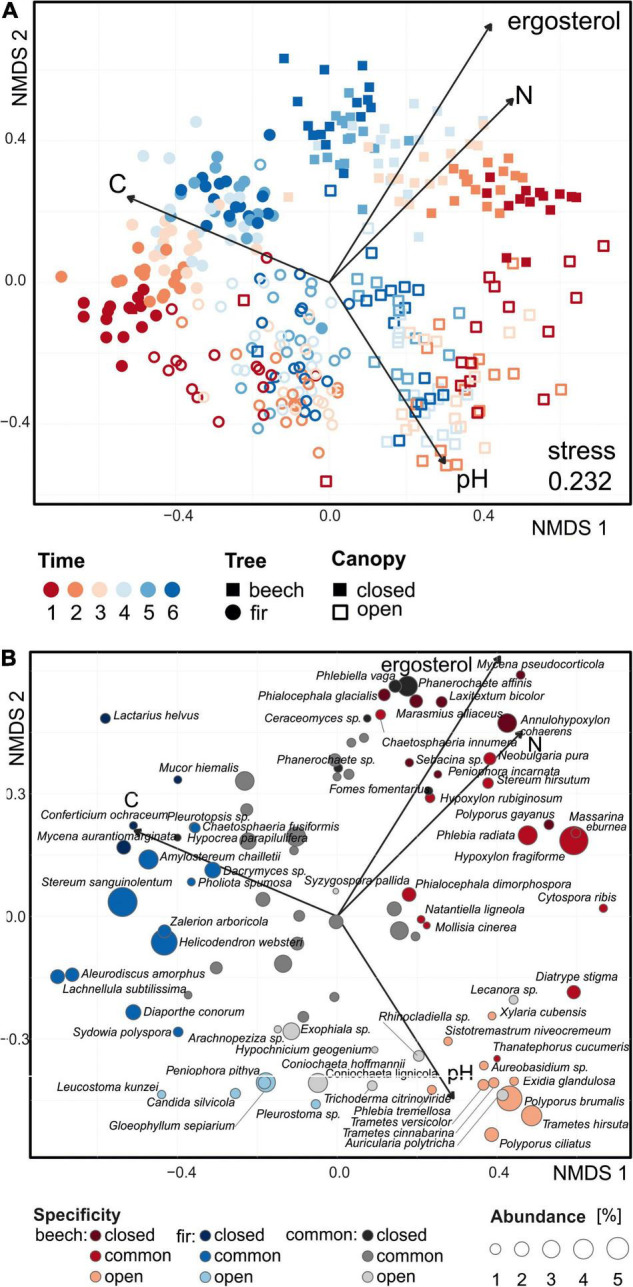
Non-metric multidimensional scaling of decomposing fine deadwood of beech and fir under different microclimatic conditions in a natural temperate forest based on dissimilarities among samples. Analysis was based on Euclidean distances on Hellinger-transformed relative abundances. Vectors indicate environmental variables with significant correlations with NMDS results (*P* < 0.05). **(A)** Samples are represented by points. OTUs with relative abundances over 0.5% in at least three samples were included. **(B)** Each point represents the abundant species and its specificity for substrate and microclimatic conditions. The size of the point is scaled based on the relative average abundances in all samples. Species with maximal abundances over 5% in at least three samples are displayed.

Based on the data of all OTUs with relative abundances over 0.5% in three or more samples, fungal communities detected in beech and fir FWD clustered separately, as well as in the case of the different canopy types (Figure 2A). Although the stress value of NMDS was high, the results were supported by other statistical results. Over time, fungal communities underwent changes but remained treatment-specific. The most important differences were detected in the initial stage of decomposition. Although the composition of communities tended to converge, treatments remained clearly separated even after 6 years of decomposition (Figure 2A). The increased diversity of deadwood origin present on site did not clearly affect the community structure. However, detailed PERMANOVA revealed a marginal effect of increased FWD diversity on fungal community structure composition in fine beech deadwood (*P* = 0.054) but not in fine fir deadwood (*P* = 0.275).

**FIGURE 3:**
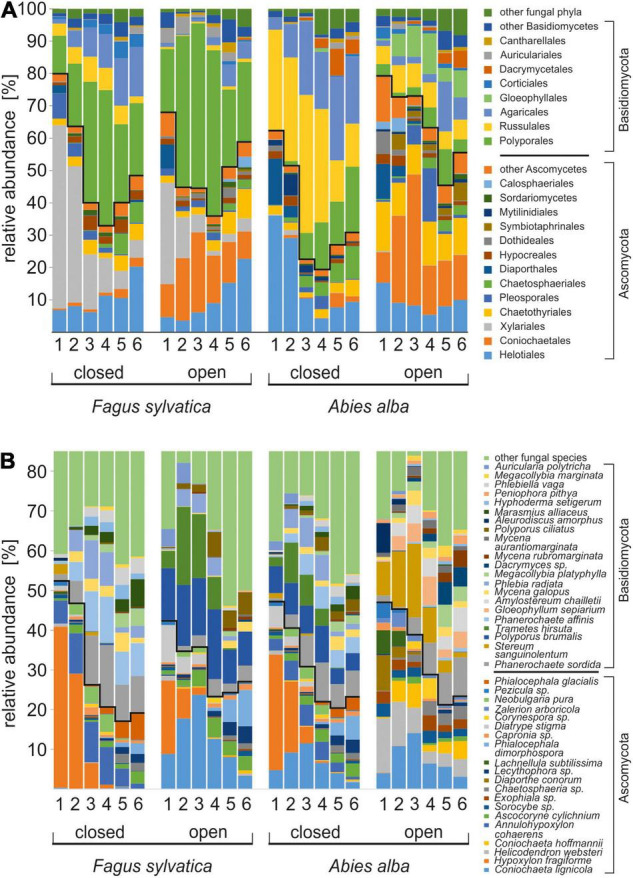
Succession of fungi in decomposing fine beech and fir deadwoods in a natural temperate forest. The data represent the means of 16 samples per treatment for fungal orders **(A)** and species **(B)**. Fungal orders and species with average abundances over 0.5% are included. Lines separate Ascomycota and Basidiomycota.

The fungal communities in fine beech and fir deadwoods were characterized by continuous changes in the relative abundances of Ascomycota and Basidiomycota. Among other fungal phyla, only Mucoromycota were more abundant in fir deadwood under the closed canopy in approximately the middle of the decomposition, where the share of their sequences was approximately 7%. The fungal community in beech FWD in year 1 was dominated by the sequences of Ascomycota (75%). With time, it initially decreased to 35% and later increased to 57% at the end of the experiment. The share of Basidiomycota sequences reached its maximum in the middle part of the experiment at 64% (Figure 3A). The share of Ascomycota was substantially higher, by 19–52% under the open canopy during the whole course of the experiment.

At the order level, beech deadwood under the closed canopy was dominated by *Xylariales*, which were gradually replaced by *Polyporales*, whereas a high share of *Coniochaetales* or *Helotiales* was observed at the end of the experiment under the open canopy (Figure 3B). Fir FWD under the closed canopy was initially dominated by *Helotiales* and *Russulales*, while the shares of *Agaricales* and *Polyporales* increased with time. In contrast, the fungal community of fir deadwood decomposing under the open canopy was phylogenetically diverse, with higher shares of *Coniochaetales*, *Chaetotriales*, *Helotiales*, *Russulales*, and *Gloephyllales* (Figure 3).

**FIGURE 4:**
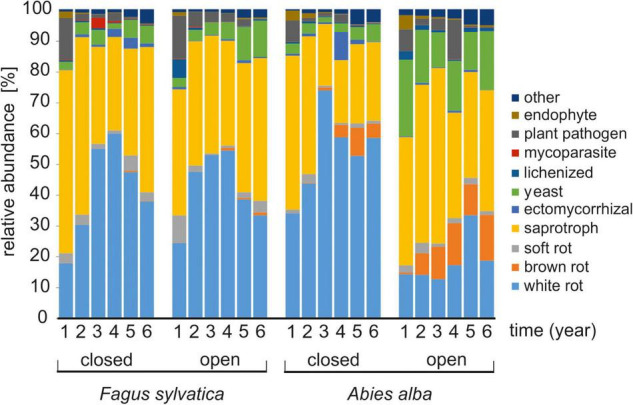
Diversity of fungal ecophysiological groups in fine beech (*Fagus sylvatica*) and fir (*Abies alba*) deadwoods decomposing in a natural temperate forest. Data represent means from 16 samples from 4 sampling sites.

Initially, both tree species were mainly colonized by fungi classified as saprotrophs (60% beech and 50% fir deadwood) with some share of white-rot fungi (18 and 34%, respectively) and plant pathogens (14 and 7%, respectively) under the closed canopy. Later, white-rot fungi dominated the decomposition of both deadwood species. Relatively higher shares of ectomycorrhizal fungi (up to 4% in beech and 9% in fir) were occasionally observed in the later phases of the experiment under a closed canopy. Brown-rot fungi increased in the later phases of fir decomposition, where their sequences represented up to 9% (Figure 4). The abundance of yeasts was clearly increased in fir deadwood under the open canopy, by an average of 2.6-fold compared to the closed canopy, and the same was observed in beech deadwood at the end of the experiment. Two-way PERMANOVA (Bray–Curtis distance, 9,999 permutations) revealed that in beech deadwood, only the time of decomposition (*P* = 0.0001) but not the canopy type (*P* = 0.1852) affected the share of fungi with different ecologies. In contrast, on fir, time (*P* = 0.0001), canopy (*P* = 0.0001) and their interaction (*P* = 0.0005) had significant effects. The increased diversity of the mixed FWD did not affect the fungi share of ecological groups.

**FIGURE 5:**
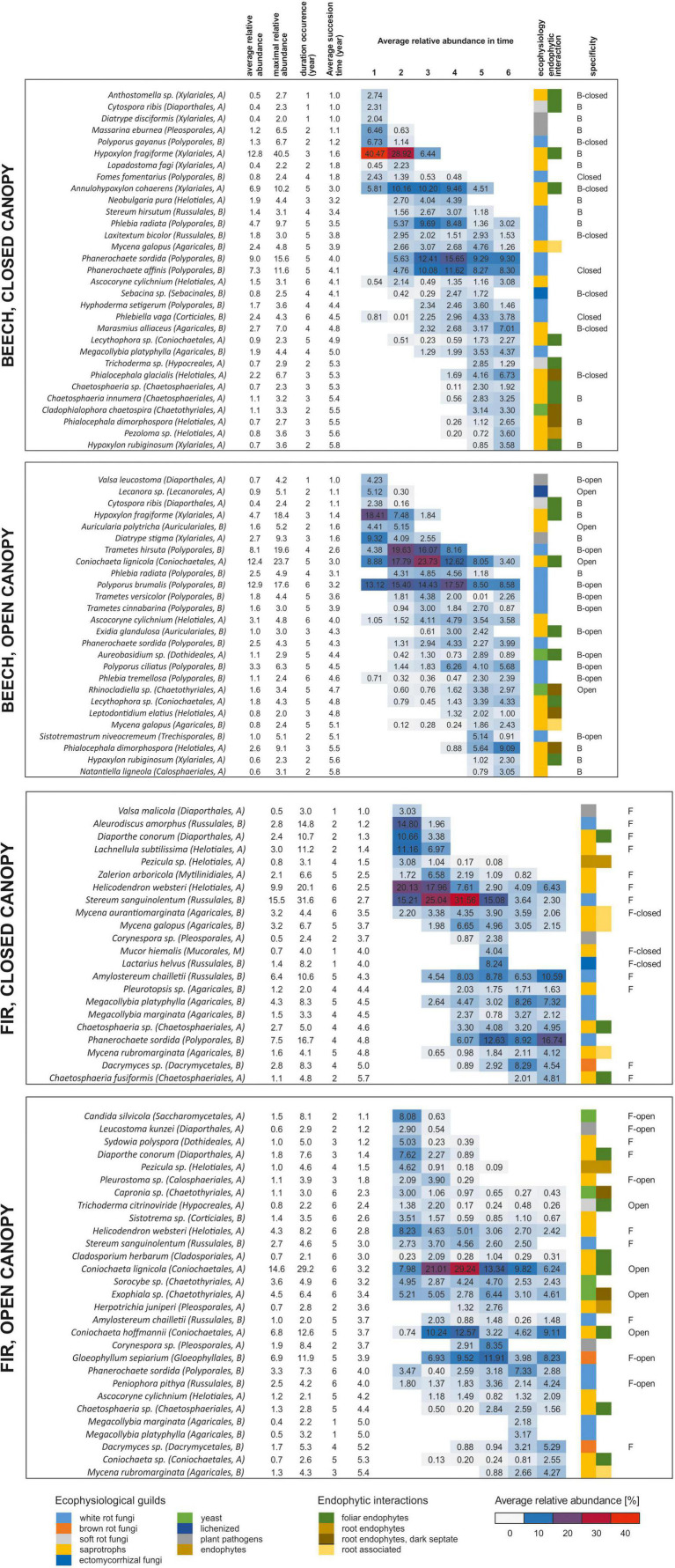
Successional development of the fungal communities in beech *(Fagus sylvatica)* and fir *(Abies alba)* fine deadwoods decomposing under closed and open canopies in a natural temperate forest. All taxa with abundances above 1% in 3 yearly observations or maximum relative abundances in any single year over 2% were considered.

**FIGURE 6:**
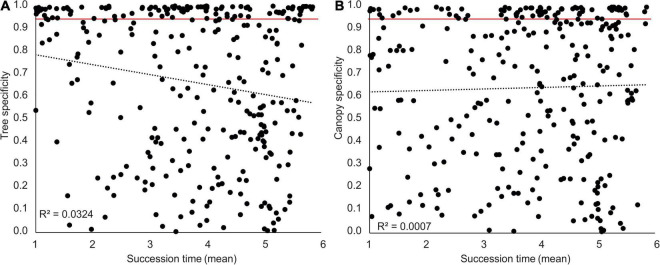
Specificity of fungal taxa to FWD type and microclimatic conditions during succession on beech (*Fagus sylvatica*) and fir (*Abies alba*) fine woody debris (FWD) in a natural temperate forest. All taxa with abundances above 0.5% in at least 3 samples and above 1% in a single sample are represented by a dot. Species were considered specific if a minimum of 95% of the detected sequences were found in one FWD type or under one of the defined microclimatic conditions (marked with full red line). Correlations between succession time (in years) and **(A)** the level of specificity to a FWD type (Spearman *R* = -0.1304, *P* = 0.0246) or **(B)** microclimatic conditions—canopy manipulation (Spearman *R* = 0. 0359, *P* = 0.5380).

The vast majority of fungal taxa were detected only over a limited timeframe in succession (Figure 5). The durations of occurrence were variable among the fungi but very often they were limited to a few years, indicating fast community turnover. Regardless of the deadwood species or canopy type, the fungi present in the initial decomposition had low occurrence durations (typically 1–2 years), while those fungi that first appeared at intermediate and late stages of the experiment were sometimes present for 4 or more years (Figure 5). Most of the highly abundant fungi were restricted to only beech deadwood (34%) or to only fir deadwood (23%). The vast majority of the fungi did not prioritize according to the canopy type (58% in beech, 73% in fir deadwood), yet as much as 19 and 10% of the fungi were closed canopy-specific in beech and fir deadwoods, respectively (Figure 2B). Tree species-specific fungi were present throughout the decomposition. However, their highest shares were observed at the beginning of decomposition, where tree-unspecific fungal species with calculated specificity <0.95 are largely missing. Canopy-specific fungi were present in equal shares over the decomposition (Figure 6). The calculated succession times of the commonly present fungal species did not significantly differ in beech or fir deadwoods, nor under open or closed canopies (Supplementary Figure 5).

Among fungi with high relative sequence abundances in beech deadwood under a closed canopy, *Hypoxylon fragiforme, Polyporus gayanus*, and *Massarina eburnea* were typical during early decomposition. Later, those and *Phanerochaete sordida, Phanerochaete affinis, Annulohypoxylon cohaerens*, and *Phlebia radiata* were replaced by *Marasmius alliaceus* and *Phialocephala glacialis* at the end of the decomposition. Decomposing fir deadwood under closed canopy was in the early phase dominated by *Helicodendron websteri, Stereum sanguinolentum, Aleurodiscus amorphus, Lachnellula subtilissima, Diaporthe conorum* followed by *Zalerion arboricola*. From the second year of decomposition, *Stereum sanguinolentum* dominated the fungal community and was later accompanied by *Amylostereum chailletii, Lactarius helvus*, and *Mycena galopus*. The late phase community typically had higher shares of sequences of *Phanerochaete sordida, Dacrymyces* sp. and *Megacollybia platyphylla.* Under the open canopy, *Hypoxylon fragiforme* is a typical fungus in the first years of decomposition, but in addition to this, *Trametes hirsuta*, *Diatrype stigma*, *Auricularia polytricha*, and *Lecanora* sp. were also found at high sequence abundances. The late phase of decomposition under the open canopy typically contained *Phialocephala dimorphospora, Polyporus ciliatus*, and *Sistotremastrum niveocremeum.* The fir deadwood under the open canopy initially showed a higher diversity of abundant fungal taxa with high shares of *Candida silvicola*, *Diaporthe conorum* and *Sydowia polyspora*. Further on, *Coniochaeta lignicola*, *Coniochaeta hoffmannii*, and *Gloeophyllum sepiarium* dominated the fungal community, along with *Corynespora* sp., *Helicodendron websteri*, and *Exophiala* sp. These taxa were replaced by *Phanerochaete sordida* and *Dacrymyces* sp. at the end of the decomposition (Figure 5).

Only a few highly abundant taxa exhibited low substrate specificity and were present in both deadwood species. This was the case for *Coniochaeta lignicola*, *Phanerochaete sordida*, *Mycena galopus*, and *Megacollybia platyphylla* (Supplementary Figure 6). The share of fungi common to both tree species increased over time and declined shortly before the end of the experiment, which was similar to the diversity of the fungal decomposer community overall. Their preferences for certain succession stages were mostly similar in beech and fir deadwoods (*P* < 0.0001, *R*^2^ = 0.6999). Similarly, the succession times of fungi without canopy specificity did not differ (*P* < 0.0001, *R*^2^ = 0.8994, beech deadwood; *P* < 0.0001, *R*^2^ = 0.6842, fir deadwood) (Figure 6).

## Discussion

### Fine Woody Debris in the Forest Ecosystem

The stock of fine woody debris present in the management zone of the Bavarian Forest NP was estimated at 5.3 t ha^–1^ (∼17.5 m^3^ ha^–1^), which is within the range previously reported in temperate broadleaf forests (Nordén et al., 2004). However, this estimated deadwood mass is considerably smaller than that estimated for natural beech-dominated forest reserves that is rich in dead tree trunks (coarse woody debris, CWD) and can have 130–300 m^3^ of woody debris per hectare (Christensen et al., 2005; Král et al., 2018). Calculated amount of FWD roughly corresponds to the recently reported quantity of coarse woody debris in European forests across all management types that reached only 11.5 m^3^ ha^–1^ (Forest Europe, 2015). This is the consequence of intensive forest management, where the total amount of deadwood is limited to 10–20% of its original mass. That is caused mainly by extracting the CWD (Müller-Using and Bartsch, 2009). Fine woody debris (with diameters < 10 cm) thus currently represents the bulk of the deadwood stock in the majority of the managed forest ecosystems in Europe. More importantly, the C flux through FWD is much faster than that through CWD. A recent analysis showed that 50% of mass from beech CWD is lost within 25–38 years in warm humid climates corresponding to the Bavarian Forest NP. The higher value is for CWD with diameters > 55 cm, while the lower value is for CWD with diameters of 10–25 cm (Přívětivý et al., 2016). The values for fir wood are comparable (Přívětivý et al., 2018). Compared to that, 50% of the mass loss of FWD is achieved considerably faster, within 7 years (Angst et al., 2018; Přívětivý et al., 2018). The FWD turnover is thus roughly 5 times faster than of CWD and we observe only one fifth of the deposited FWD over the time equal to lifespan of CWD. Therefore, the stock of 5.3 t ha^–1^ of FWD recorded in the Bavarian Forest NP would thus correspond, in terms of yearly production, to a stock of 26.5 t ha^–1^ (or approximately 90 m^3^ ha^–1^) of CWD, which is approximately half of the values observed in unmanaged forests (Král et al., 2010). Undoubtedly, the contribution of FWD to the flow of complex C compounds and other nutrients temporarily stored in wood into soil is highly important.

### Fine Woody Debris Decomposition

Our experiment allowed us to analyze the FWD decomposition until the branches at certain locations disintegrated and thus it covered a large proportion of the FWD lifespan. The rapid decay progress was demonstrated by the increasing ergosterol content, deadwood moisture and wood pH decrease, both of which reflect the activity of fungal wood decomposers (Baldrian et al., 2016; Lepinay et al., 2021a,b; Přívětivý and Šamonil, 2021). The higher ergosterol contents and moisture of beech FWD suggest their faster decomposition, as already described for identical tree species (Přívětivý et al., 2016; Kahl et al., 2017; Lepinay et al., 2021a; Přívětivý and Šamonil, 2021). The tree specificity of deadwood moisture was previously described for *A. abies* and *F. sylvatica* (Přívětivý and Šamonil, 2021). The tree specificity in deadwood moisture may reflect the different retention capacity of deadwood based on its physiochemical properties. The rapid advance of beech FWD decomposition was also indicated by the presence of ectomycorrhizal species that typically occur in the latest stages of succession (Rajala et al., 2012; Baldrian et al., 2016).

The consistent changes in deadwood chemistry reflected its initial composition and set the stage for a functionally even decomposition process regardless of the deadwood origin and microclimatic conditions. In detail, the open canopy slowed initial fungal colonization, as demonstrated by the significantly lower amounts of ergosterol in year 1 and slower acidification, but this canopy effect disappeared later. The changing pH of the FWD was likely one of the factors affecting fungal community assembly since it is a strong predictor of fungal community composition on deadwood (Purahong et al., 2018), and many deadwood fungi show specific pH preferences (Lepinay et al., 2021a).

### Fungal Community Composition and Its Drivers

The compositions of the fine deadwood fungal communities were strongly affected by both tree species and microclimatic conditions. Our results are thus in line with observations on coarse deadwood that identified substrate quality (tree species) and climate (temperature and moisture) as the key factors affecting fungal communities (Rayner and Boddy, 1988; Rajala et al., 2012; Abrego et al., 2017; Bani et al., 2018; Purahong et al., 2018; Harmon et al., 2020). Beyond the seasonal climate, microclimate extremes cause more variability in the environment. Importantly, both the tree species and microclimatic conditions influenced the whole successional development of fine woody debris fungal communities, thus confirming the sensitivity of the FWD fate to microclimates.

Fungal communities on beech and fir FWD underwent successional development but remained distinct throughout the whole experiment. Previously, certain fungal species were reported to prefer beech or fir CWD in mixed forests (Lepinay et al., 2021a), but the differences were often indistinct (Baldrian et al., 2016). The level of tree species specificity on FWD observed here was considerably higher. The observed specialization of Ascomycota to FWD or deadwood of smaller size is in line with previous reports (Nordén et al., 2004; Rajala et al., 2012). The colonization of FWD is certainly easier than that of CWD due to its large surface-to-volume ratio and allows the establishment of Ascomycota, whose ability to decompose recalcitrant wood components is limited (Eichlerová et al., 2015). The high share of Ascomycota in the initial phase of deadwood and litter decomposition is typical (Voříšková and Baldrian, 2013; Baldrian et al., 2016; Štursová et al., 2020; Lepinay et al., 2021a) and reflects the high share of opportunists in this fungal phylum that prefer to utilize the less recalcitrant components of the plant biomass (Algora Gallardo et al., 2021). Detection of Mucoromycota in the fourth year of decomposition may indicate their involvement in the decomposition of the mycelia of early fungal colonizers since these fungi are frequently associated with decomposing fungal biomass (Brabcová et al., 2018; Algora Gallardo et al., 2021).

In both deadwood species, the initial community of saprotrophs, white-rot fungi and plant pathogens was later replaced and dominated by various white-rot fungi. Similar to our finding, brown-rot fungi are most frequently found in the intermediate stage of CWD decomposition (Rajala et al., 2012). Brown-rot fungi are considered to have adapted to decompose wood where there is lower competitive pressure (Song et al., 2017), which is clearly contrasting with the many cord-forming white-rot soil fungi with combative life-history strategies (Rayner and Boddy, 1988; Boddy and Hiscox, 2017). We found that white-rot fungi were commonly present on both substrates. Ectomycorrhizal fungi were more represented only in the later decomposition of FWD. They are frequently found on tree seedlings commonly growing on dead tree trunks and their low frequency in FWD is likely due to the fact that this is impossible on FWD.

Experimental opening of the canopy in our experiment resulted in considerably different penetration rates of solar radiation, which were, on average, sevenfold higher in cleared plots on an area of 0.5 ha (Krah et al., 2018), and the summer temperatures on deadwood surfaces were on average twofold higher under open canopies than under closed canopies (Müller et al., 2020). The dense canopy acts as a thermal buffer, possibly mitigating the severity of the impacts of climate change on forest biodiversity and functioning (De Frenne et al., 2019). Canopy gaps can promote the diversity of wood-inhabiting fungi on CWD (Brazee et al., 2014) as well as arthropods (Horák et al., 2016; Seibold et al., 2018). An increase in fungal diversity was observed only in the fir FWD in the present study, but the open canopy clearly prioritize different FWD fungi than the closed canopy. We assume that fungal taxa presence is driven by a combination of nutritional and climatic factors. Interestingly, the fir FWD decomposition under the harsh open canopy environment was dominated by Ascomycota throughout the whole process (Figure 3). Some ascomycetes are endophytes or pioneer species that can rapidly colonize new and competition-free woody substrates (Menkis et al., 2004; Parfitt et al., 2010). Their presence may indicate lower competitive forces being present in fir FWD. Broad niche differentiation in FWD (Bässler et al., 2010) is further supported by the diversity of fungal guilds detected in canopy gaps, with a high abundance of plant pathogens, yeasts (in both FWD types) and brown-rot and soft-rot fungi observed in fir (Figure 4). This may also indicate a lower ability of otherwise highly competitive white-rot fungi to cope with stressful environmental conditions leading to the phylogenetic diversification of the fungal community.

In general, the guild of foliar endophytes was very abundant in the early decomposition regardless of the tree species, which confirms that they are priority colonizers initiating wood decomposition (Song et al., 2017). As already proposed, there is a strong priority effect in fungal community assembly in deadwood (Boddy, 2000; Hiscox et al., 2015; Krah et al., 2018). An increased abundance of yeasts was typical for advanced decomposition. These fungi are known to utilize various substrates (Mašínová et al., 2018), including fungal mycelium (Brabcová et al., 2018). A high proportion of the recorded fungi were dark septate endophytes recently detected in decomposing roots (Kohout et al., 2021), a type of substrate that is similar in size and composition to FWD.

Individual taxa showed various levels of preferences for tree species, microclimatic conditions and certain decomposition stages. Regardless of the conditions, fungal species were typically present over only a limited timeframe, typically 1–2 years for the early colonizers and a longer time for later inhabitants. Such a fast turnover of taxa is rather typical of litter (Voříšková and Baldrian, 2013; Urbanová et al., 2015) and less pronounced on coarse deadwood, where many fungal taxa persist for a long time during the deadwood lifecycle (Baldrian et al., 2016). Fungal taxa associated with both beech and fir appeared at a similar timepoint, demonstrating that the succession time of common taxa is an equally stable trait of FWD-associated fungi as that for litter decomposers (Štursová et al., 2020). Tree-specific fungi were detected over the whole decomposition and were most abundant during the initial decomposition. This is likely a consequence of the tree specificity of fungal endophytes and their priority in colonization or a result of filtering by nutrient availability (Bhatnagar et al., 2018). The microclimatic conditions seem to be a weaker filter of the initial colonization, and canopy-specific fungi were rather evenly distributed in time with a slightly higher share in later decomposition.

The contribution of the FWD to the forest biodiversity pool has not been frequently addressed. Forest fungal biodiversity is broadly limited by deadwood manipulation during forest management (Juutilainen et al., 2014), which also includes FWD manipulation (Sandström et al., 2019). Follow up studies emphasized, that particularly CWD of a smaller diameter is highly important in the preservation of fungal biodiversity in managed forests (Heilmann-Clausen and Christensen, 2004). Nonetheless, the very fine (<5 cm diameter) and fine woody debris (5–10 cm diameter) may harbor 75% of the total fungal diversity (Abrego et al., 2017; Juutilainen et al., 2017). Comparing our results with an equally detailed study of fungal communities that are important in the CWD decomposition of *Fagus sylvaticus* and *Abies alba* (Lepinay et al., 2021b), we enumerated identical overall numbers of fungal OTUs. We confirmed that FWD is very important for the diversity of Ascomycota, whereas CWD must also be present to ensure the occurrence of many basidiomycete species (Nordén et al., 2004). The assemblage of CWD fungal communities is mainly determined by tree species and deadwood stage (Müller et al., 2020), while we stress that microclimatic factors are another key determinant of FWD fungal biodiversity.

## Conclusion

This study highlights the importance of FWD in forest carbon stock, biodiversity and high variability of microclimatic conditions in mitigating the impact of conventional forest management. In agreement with our hypothesis, tree species were an important driver of fungal community assembly in FWD. Microclimatic conditions represented another, almost equally strong determinant of the fungal community. Combinations of these factors resulted in typical succession series during FWD decomposition. Increased deadwood species diversity did not affect the fungal communities of unique FWDs. Ascomycota were the main fungal group involved in FWD decomposition. Depending on tree species and environmental factors, these fungi may dominate the whole decomposition process in contrast to the dominant role of Basidiomycota in coarse deadwood. Tree-specific fungal species were common during initial decomposition, whereas canopy-specific fungi were less frequent and rather evenly distributed over time. The presence of a sufficient amount of FWD, forest stand diversity and microclimatic conditions are important factors in the maintenance of fungal diversity. Moreover, fine woody debris represents an important input of recalcitrant plant biomass whose decomposition liberates wood nutrients into soil. Fine deadwood production is comparable in size to that of coarse deadwood in natural forests and represents the bulk of deadwood turnover in the majority of European forests that are managed and thus devoid of coarse deadwood.

## Data Availability Statement

The datasets presented in this study can be found in online repositories. The names of the repository/repositories and accession number(s) can be found below: https://www.ncbi.nlm.nih.gov/sra, PRJNA671809.

## Author Contributions

CB, PB, JM, and RB: conceptualization. CB, VT, PZ, PB, and VB: experimental design and methodology. VT, PZ, IE, CL, MŠ, and VB: performance of experimental work, data evaluation, and statistical analyses. CB, PB, and VB: validation. VB: writing—original draft preparation. VT, CL, CB, JM, PB, and VB: writing—review and editing. PB and VB: supervision, project administration, and funding acquisition. All authors have read and agreed to the published version of the manuscript.

## Conflict of Interest

The authors declare that the research was conducted in the absence of any commercial or financial relationships that could be construed as a potential conflict of interest.

## Publisher’s Note

All claims expressed in this article are solely those of the authors and do not necessarily represent those of their affiliated organizations, or those of the publisher, the editors and the reviewers. Any product that may be evaluated in this article, or claim that may be made by its manufacturer, is not guaranteed or endorsed by the publisher.

## Footnotes

1
https://www.nhm.uio.no/english/research/infrastructure/past/

## Supplementary Material

Supplementary Figure 1 Experimental design, based on a figure from Krah et al. (2018).

Supplementary Figure 2 Amount of deadwood present on the forest floor of a Bavarian Forest NP and moisture levels of the fine woody debris (FWD), log scale. Small FWD: 0.5–1.5 cm in diameter, mid-size FWD: 1.6–5.0 cm in diameter, large FWD: 5.1–10.0 cm in diameter, parts with minimum lengths of 10 cm. Boxes indicate the lower and upper quartiles, and individual points represent separate 2 × 2 m sampling plots.

Supplementary Figure 3 Diversity of fungi during the decomposition of the FWD of beech and fir in a temperate natural forest expressed as OTU richness and Chao 1 estimates. Development of diversity estimated over time (left), overall differences not considering community development over time (right).

Supplementary Figure 4 Venn diagram representing the results of variation partitioning analyses on Hellinger-transformed OTU abundances. **(A)** Tree species and canopy cover calculated using all FWD samples, **(B)** canopy cover, time and deadwood chemism for beech FWD, C: canopy, time and deadwood chemism for fir FWD.

Supplementary Figure 5 Occurrence of fungal taxa during succession on beech and fir fine woody debris (FWD) in a montane forest according to FWD type and microclimatic conditions. Correlations between succession time (in years) of each fungal taxon present on mixed beech and fir FWD (**A**, *P* < 0.0001), detected under open and closed canopies in beech (**B**, *P* < 0.0001) or fir (**C**, *P* < 0.0001) fine woody debris. All taxa with abundances above 0.5% in three or more samples and above 1% in at least one sample were considered.

Supplementary Figure 6 Commonly present fungal species and their succession during fine woody debris decomposition. The figure includes species present in at least three samples over 1%, with maximal abundance in a single year over 2% or an average abundance over 0.5%. **(A)** Fungal species common in beech and fir deadwoods, **(B)** beech deadwood, both canopy types, **(C)** fir deadwood, both canopy types.
