# Supplementary material for: Fungal Community Development in Decomposing Fine Deadwood Is Largely Affected by Microclimate
Source: Front Microbiol. 2022 Apr 13;13:835274. doi: 10.3389/fmicb.2022.835274 (PMC9045801; doi:10.3389/fmicb.2022.835274)
Supplement: Supplementary Figure 1 — Experimental design, based on a figure from Krah et al. (2018). [file Data_Sheet_6.PDF]

BEECH / FIR , all samples

Species	ecophysiology	endophytic	specificity	Succesion time mean [year]	average abundance [%]	average abundance (whole decomposition) [%]	maximal year average abundance [%]	duration of occurrence	1	2	3	4	5	6	average abundance (whole decomposition) [%]	maximal year average abundance [%]	duration of occurrence	1	2	3	4	5	6
Lecanora sp. (Lecanorales, A)			open	1.1	0.3	0.5	2.7	2	2.7	0.2					0.0	0.2	2	0.2	0.0				
Auricularia polytricha (Auriculariales, B)			open	1.7	0.5	0.8	2.7	2	2.3	2.7					0.2	0.9	2	0.1	0.9				
Pezicula sp. (Helotiales, A)				1.9	0.6	0.3	1.1	6	1.1	0.0	0.0	0.1	0.2	0.2	0.9	3.9	4	3.9	1.0	0.2	0.1		
Coniochaeta lignicola (Coniochaetales, A)			open	3.1	6.8	6.3	11.5	5	4.8	9.2	11.5	6.6	4.0		7.4	14.1	6	4.0	10.8	14.1	6.5	5.6	3.1
Sorocybe sp. (Chaetothyriales, A)				3.2	1.4	0.5	0.9	6	0.9	0.5	0.3	0.1	0.2	0.8	2.4	3.3	6	3.3	1.9	2.4	3.1	1.7	1.9
Capronia sp. (Chaetothyriales, A)				3.2	0.8	0.9	1.4	6	0.4	0.5	1.4	0.8	0.9	1.1	0.8	1.9	6	1.9	0.9	0.6	0.8	0.3	0.4
Exophiala sp. (Chaetothyriales, A)			open	3.6	1.3	0.2	0.3	6	0.1	0.2	0.3	0.1	0.1	0.3	2.5	3.9	6	3.1	2.7	1.4	3.9	1.7	2.4
Corynespora sp. (Pleosporales, A)				3.7	0.7	0.3	0.9	2			0.6	0.9			1.2	5.1	2			1.9	5.1		
Coniochaeta hoffmannii (Coniochaetales, A)			open	3.9	1.8	0.2	0.4	6	0.1	0.3	0.1	0.2	0.2	0.4	3.5	6.1	5		5.3	6.1	1.6	2.9	4.6
Mycena galopus (Agaricales, B)				4.0	1.8	1.6	3.4	5		1.3	1.7	1.4	3.4	1.9	2.0	3.7	6	0.8	1.3	3.7	2.7	2.3	1.6
Ascocoryne cylichnium (Helotiales, A)				4.0	1.6	2.3	3.3	6	0.8	1.8	2.2	3.1	2.3	3.3	0.8	1.7	5		0.8	1.7	0.5	0.9	1.2
Phanerochaete sordida (Polyporales, B)				4.3	5.6	5.7	9.8	5		3.4	7.8	9.8	6.0	6.6	5.4	9.8	6	1.7	0.5	4.4	8.0	8.1	9.8
Hyphoderma setigerum (Polyporales, B)				4.4	0.7	0.9	1.9	4			1.2	1.4	1.9	0.7	0.4	0.8	5		0.3	0.5	0.4	0.8	0.6
Phanerochaete affinis (Polyporales, B)			closed	4.4	2.0	3.7	5.6	5		2.3	5.2	5.6	4.4	4.0	0.4	0.9	4			0.4	0.4	0.7	0.9
Megacollybia marginata (Agaricales, B)				4.6	0.6	0.3	0.6	4			0.2	0.5	0.6	0.2	0.9	2.7	4			1.2	0.4	2.7	1.1
Lecythophora sp. (Coniochaetales, A)				4.8	0.9	1.3	3.3	5		0.7	0.3	1.0	2.5	3.3	0.4	1.0	6	0.2	0.1	0.1	0.1	1.0	0.9
Megacollybia platyphylla (Agaricales, B)				4.8	1.7	1.0	2.5	4			0.7	1.0	2.5	2.1	2.4	5.6	5		1.3	2.3	1.6	5.6	3.7
Chaetosphaeria sp. (Chaetosphaeriales, A)				5.0	1.3	0.6	1.7	2					1.7	1.7	2.0	3.3	5		0.4	1.8	3.3	2.9	3.3
Mycena rubromarginata (Agaricales, B)				5.0	0.9	0.2	0.6	4			0.2	0.1	0.6	0.5	1.5	4.2	4			0.5	1.4	2.4	4.2
Pezoloma sp. (Helotiales, A)				5.1	0.3	0.5	2.2	3				0.1	0.4	2.2	0.0	0.1	6	0.0	0.0	0.0	0.0	0.0	0.1
Phlebiella vaga (Corticiales, B)			closed	5.2	0.7	1.2	2.6	6	0.4	0.0	1.2	1.4	2.6	1.8	0.1	0.4	2					0.0	0.4
Cladophialophora chaetospira (Chaetothyriales, A)				5.5	0.4	0.7	2.1	2					1.9	2.1	0.1	0.4	2					0.2	0.4

BEECH, canopy closed / open

Species	ecophysiology	endophytic	specificity	Succesion time mean [year]	average abundance [%]	average abundance (whole decomposition) [%]	maximal year average abundance [%]	duration of occurrence	1	2	3	4	5	6	average abundance (whole decomposition) [%]	maximal year average abundance [%]	duration of occurrence	1	2	3	4	5	6
Cytospora ribis (Diaporthales, A)			B	1.0	0.4	0.4	2.3	1	2.3						0.4	2.4	2	2.4	0.2				
Massarina eburnea (Pleosporales, A)			B	1.0	0.7	1.2	6.5	2	6.5	0.6					0.1	0.7	1	0.7					
Diatrype disciformis (Xylariales, A)			B	1.1	0.2	0.4	2.0	1	2.0						0.1	0.5	2	0.5	0.1				
Hypoxylon fragiforme (Xylariales, A)			B	1.5	8.7	12.8	40.5	3	40.5	28.9	6.4				4.7	18.4	3	18.4	7.5	1.8			
Diatrype stigma (Xylariales, A)			B	1.5	1.5	0.3	1.0	3	1.0	0.4	0.0				2.7	9.3	3	9.3	4.1	2.5			
Lopadostoma fagi (Xylariales, A)			B	1.8	0.3	0.4	2.2	2	0.4	2.2					0.1	0.4	2	0.2	0.4				
Neobulgaria pura (Helotiales, A)			B	2.9	1.2	1.9	4.4	3		2.7	4.0	4.4			0.5	1.4	3		1.4	1.3	0.4		
Stereum hirsutum (Russulales, B)			B	3.3	0.8	1.4	3.1	4		1.6	2.7	3.1	1.2		0.2	0.7	4	0.1	0.2	0.2	0.7		
Phlebia radiata (Polyporales, B)			B	3.3	3.6	4.7	9.7	5		5.4	9.7	8.5	1.4	3.0	2.5	4.9	4		4.3	4.9	4.6	1.2	
Capronia sp. (Chaetothyriales, A)				3.9	0.9	0.8	2.0	6	0.3	0.7	2.0	0.6	0.7	0.7	0.9	1.5	6	0.6	0.3	0.8	1.0	1.1	1.5
Ascocoryne cylichnium (Helotiales, A)				4.1	2.3	1.5	3.1	6	0.5	2.1	0.5	1.3	1.2	3.1	3.1	4.8	6	1.0	1.5	4.1	4.8	3.5	3.6
Phanerochaete sordida (Polyporales, B)				4.1	5.7	9.0	15.6	5		5.6	12.4	15.6	9.3	9.3	2.5	4.3	5		1.3	2.9	4.3	2.3	4.0
Mycena galopus (Agaricales, B)				4.5	1.6	2.4	4.8	5		2.7	3.1	2.7	4.8	1.3	0.8	2.4	5		0.1	0.3	0.2	1.9	2.4
Leptodontidium elatius (Helotiales, A)				4.7	0.5	0.2	0.4	5		0.2	0.0	0.1	0.3	0.4	0.8	2.0	3				1.3	2.0	1.0
Lecythophora sp. (Coniochaetales, A)				4.9	1.4	0.9	2.3	5		0.5	0.2	0.6	1.7	2.3	1.8	4.3	5		0.8	0.5	1.4	3.4	4.3
Megacollybia platyphylla (Agaricales, B)				5.0	1.0	1.9	4.4	4			1.3	2.0	3.5	4.4	0.2	1.2	1					1.2	
Natantiella ligneola (Calosphaeriales, A)			B	5.4	0.3	0.0	0.1	3				0.1	0.1	0.1	0.6	3.1	2					0.8	3.1
Chaetosphaeria sp. (Chaetosphaeriales, A)				5.4	0.6	0.7	2.3	3				0.1	2.3	1.9	0.4	1.4	2					1.0	1.4
Chaetosphaeria innumera (Chaetosphaeriales, A)			B	5.5	0.7	1.1	3.2	3				0.6	2.8	3.2	0.3	1.1	2					0.4	1.1
Phialocephala dimorphospora (Helotiales, A)			B	5.5	1.6	0.7	2.7	3				0.3	1.1	2.7	2.6	9.1	3				0.9	5.6	9.1
Cladophialophora chaetospira (Chaetothyriales, A)				5.6	0.7	1.1	3.3	2					3.1	3.3	0.2	0.9	2					0.4	0.9
Hypoxylon rubiginosum (Xylariales, A)			B	5.7	0.7	0.7	3.6	2					0.9	3.6	0.6	2.3	2					1.0	2.3
Pezoloma sp. (Helotiales, A)				5.7	0.5	0.8	3.6	3				0.2	0.7	3.6	0.2	0.9	2					0.1	0.9

FIR, canopy closed / open

Species	ecophysiology	endophytic	specificity	Succesion time mean [year]	average abundance [%]	average abundance (whole decomposition) [%]	maximal year average abundance [%]	duration of occurrence	1	2	3	4	5	6	average abundance (whole decomposition) [%]	maximal year average abundance [%]	duration of occurrence	1	2	3	4	5	6
Sydowia polyspora (Dothideales, A)			F	1.2	0.6	0.3	1.6	2	1.6	0.1					1.0	5.0	3	5.0	0.2	0.4			
Diaporthe conorum (Diaporthales, A)			F	1.4	2.1	2.4	10.7	2	10.7	3.4					1.8	7.6	3	7.6	2.3	0.9			
Aleurodiscus amorphus (Russulales, B)			F	1.4	1.6	2.8	14.8	2	14.8	2.0					0.3	0.9	2	0.7	0.9				
Lachnellula subtilissima (Helotiales, A)			F	1.5	1.7	3.0	11.2	2	11.2	7.0					0.3	1.0	2	0.9	1.0				
Pezicula sp. (Helotiales, A)				1.5	0.9	0.8	3.1	4	3.1	1.0	0.2	0.1			1.0	4.6	4	4.6	0.9	0.2	0.1		
Capronia sp. (Chaetothyriales, A)				2.6	0.8	0.5	0.8	6	0.7	0.8	0.3	0.5	0.3	0.4	1.1	3.0	6	3.0	1.1	1.0	0.6	0.3	0.4
Helicodendron websteri (Helotiales, A)			F	2.7	7.1	9.9	20.1	6	20.1	18.0	7.6	2.9	4.1	6.4	4.3	8.2	6	8.2	4.6	5.0	3.1	2.7	2.4
Stereum sanguinolentum (Russulales, B)			F	2.8	9.1	15.5	31.6	5	15.2	25.0	31.6	15.1	3.6		2.7	4.6	5	2.7	3.7	4.6	2.6	2.5	
Zalerion arboricola (Mytilinidiales, A)			F	3.1	1.4	2.1	6.6	5	1.7	6.6	2.2	1.1	0.8		0.7	1.1	6	0.7	0.6	0.6	0.5	0.7	1.1
Sorocybe sp. (Chaetothyriales, A)				3.4	2.3	1.0	1.4	6	1.4	0.8	0.7	1.1	0.7	1.4	3.6	4.9	6	4.9	2.9	4.2	4.7	2.5	2.4
Cladosporium herbarum (Cladosporiales, A)				3.4	0.4	0.1	0.2	5		0.0	0.2	0.2	0.1	0.0	0.7	2.1	6	0.2	2.1	0.3	1.0	0.3	0.3
Herpotrichia juniperi (Pleosporales, A)				3.7	0.5	0.2	0.9	2			0.4	0.9			0.7	2.8	2			1.3	2.8		
Corynespora sp. (Pleosporales, A)				3.7	1.2	0.5	2.4	2			0.9	2.4			1.9	8.4	2			2.9	8.4		
Mycena galopus (Agaricales, B)				3.8	2.0	3.2	6.7	5		2.0	6.7	5.0	3.0	2.1	0.8	1.6	6	0.8	0.6	0.4	0.4	1.6	1.1
Ascocoryne cylichnium (Helotiales, A)				3.8	0.8	0.5	1.9	5		0.4	1.9	0.2	0.4	0.3	1.2	2.1	5		1.2	1.5	0.8	1.3	2.1
Amylostereum chailletii (Russulales, B)			F	4.0	3.7	6.4	10.6	5		4.5	8.0	8.8	6.5	10.6	1.0	2.0	5		2.0	0.9	1.5	0.3	1.5
Phanerochaete sordida (Polyporales, B)				4.4	5.4	7.5	16.7	4			6.1	12.6	8.9	16.7	3.3	7.3	6	3.5	0.4	2.6	3.2	7.3	2.9
Chaetosphaeria sp. (Chaetosphaeriales, A)				4.5	2.0	2.7	5.0	4			3.3	4.1	3.2	5.0	1.3	2.8	5		0.5	0.2	2.8	2.6	1.6
Pleurotopsis sp. (Agaricales, B)			F	4.6	0.7	1.2	2.0	4			2.0	1.7	1.7	1.6	0.2	1.0	3				0.1	1.0	
Megacollybia marginata (Agaricales, B)				4.7	0.9	1.5	3.3	4			2.4	0.8	3.3	2.1	0.4	2.2	1					2.2	
Megacollybia platyphylla (Agaricales, B)				4.8	2.4	4.3	8.3	5		2.6	4.5	3.0	8.3	7.3	0.5	3.2	1					3.2	
Coniochaeta sp. (Coniochaetales, A)				4.9	0.7	0.6	1.3	5		0.8	0.3	0.1	1.3	1.3	0.7	2.6	5		0.1	0.2	0.2	0.8	2.6
Cladophialophora sp. (Chaetothyriales, A)				4.9	0.6	0.5	1.8	5		0.1	0.1	0.3	0.9	1.8	0.6	1.4	6	0.3	0.2	0.3	0.5	1.2	1.4
Dacrymyces sp. (Dacrymycetales, B)			F	5.1	2.3	2.8	8.3	4			0.9	2.9	8.3	4.5	1.7	5.3	4			0.9	0.9	3.2	5.3
Mycena rubromarginata (Agaricales, B)				5.1	1.5	1.6	4.1	5		0.6	1.0	1.8	2.1	4.1	1.3	4.3	3				0.9	2.7	4.3
Chaetosphaeria fusiformis (Chaetosphaeriales, A)			F	5.7	0.7	1.1	4.8	2					2.0	4.8	0.3	1.0	2					0.5	1.0

Ecophysiological guilds

- white rot fungi
- brown rot fungi
- soft rot fungi
- saprotrophs
- ectomycorrhizal fungi
- yeast
- lichenized
- plant pathogens
- endophytes

Endophytic interactions

- foliar endophytes
- root endophytes
- root endophytes, dark septate
- root associated

Average relative abundance [%]

0 10 20 30 40
