# Supplementary material for: Fungal Community Development in Decomposing Fine Deadwood Is Largely Affected by Microclimate
Source: Front Microbiol. 2022 Apr 13;13:835274. doi: 10.3389/fmicb.2022.835274 (PMC9045801; doi:10.3389/fmicb.2022.835274)
Supplement: Supplementary Figure 2 — Amount of deadwood present on the forest floor of a Bavarian Forest NP and moisture levels of the fine woody debris (FWD), log scale. Small FWD: 0.5–1.5 cm in diameter, mid-size FWD: 1.6–5.0 cm in diameter, large FWD: 5.1–10.0 cm in diameter, parts with minimum lengths of 10 cm. Boxes indicate the lower and upper quartiles, and individual points represent separate 2 × 2 m sampling plots. [file Data_Sheet_1.PDF]

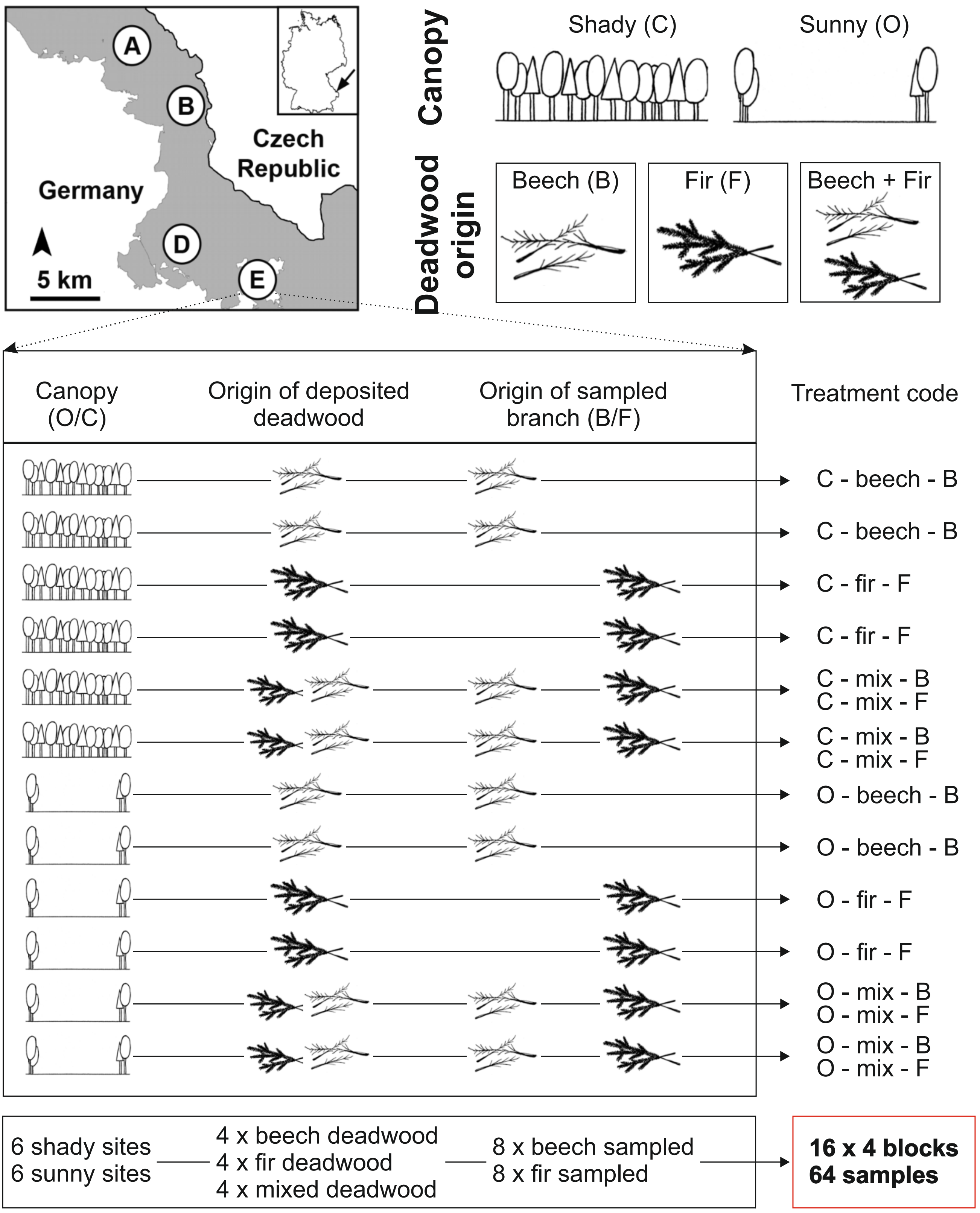

A
B
Czech Republic
Germany
D
E
5 km
Canopy
Shady (C)
Sunny (O)
Deadwood origin
Beech (B)
Fir (F)
Beech + Fir
Canopy (O/C)
Origin of deposited deadwood
Origin of sampled branch (B/F)
Treatment code
C - beech - B
C - beech - B
C - fir - F
C - fir - F
C - mix - B
C - mix - F
C - mix - B
C - mix - F
O - beech - B
O - beech - B
O - fir - F
O - fir - F
O - mix - B
O - mix - F
O - mix - B
O - mix - F
6 shady sites
6 sunny sites
4 x beech deadwood
4 x fir deadwood
4 x mixed deadwood
8 x beech sampled
8 x fir sampled
16 x 4 blocks
64 samples
