# Supplementary figures and images for: Fungal Community Development in Decomposing Fine Deadwood Is Largely Affected by Microclimate
Source: Front Microbiol. 2022 Apr 13;13:835274. doi: 10.3389/fmicb.2022.835274 (PMC9045801; doi:10.3389/fmicb.2022.835274)

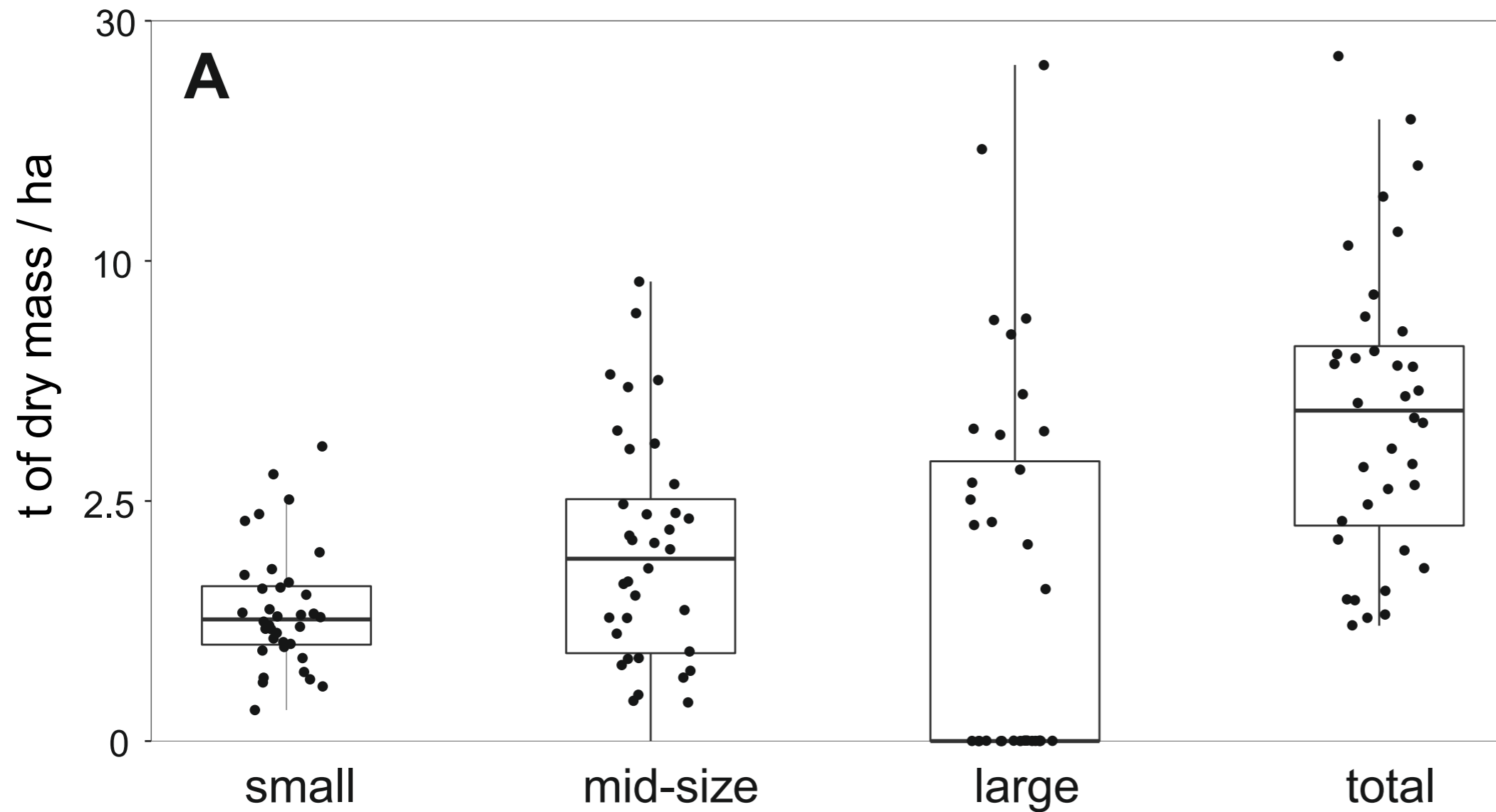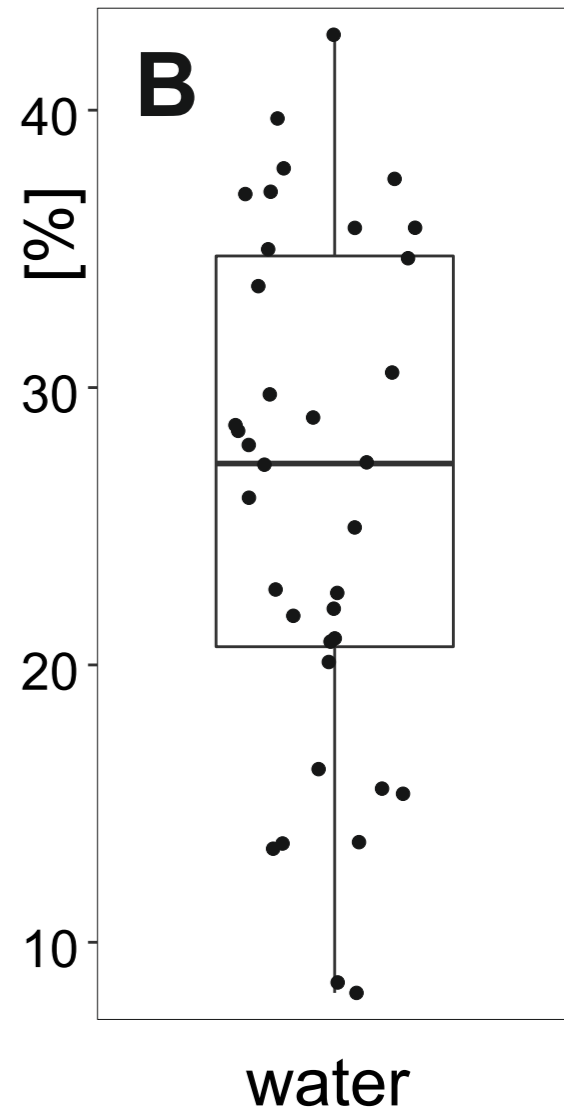

Supplement: Supplementary Figure 3 — Diversity of fungi during the decomposition of the FWD of beech and fir in a temperate natural forest expressed as OTU richness and Chao 1 estimates. Development of diversity estimated over time (left), overall differences not considering community development over time (right). [file Data_Sheet_2.PDF]

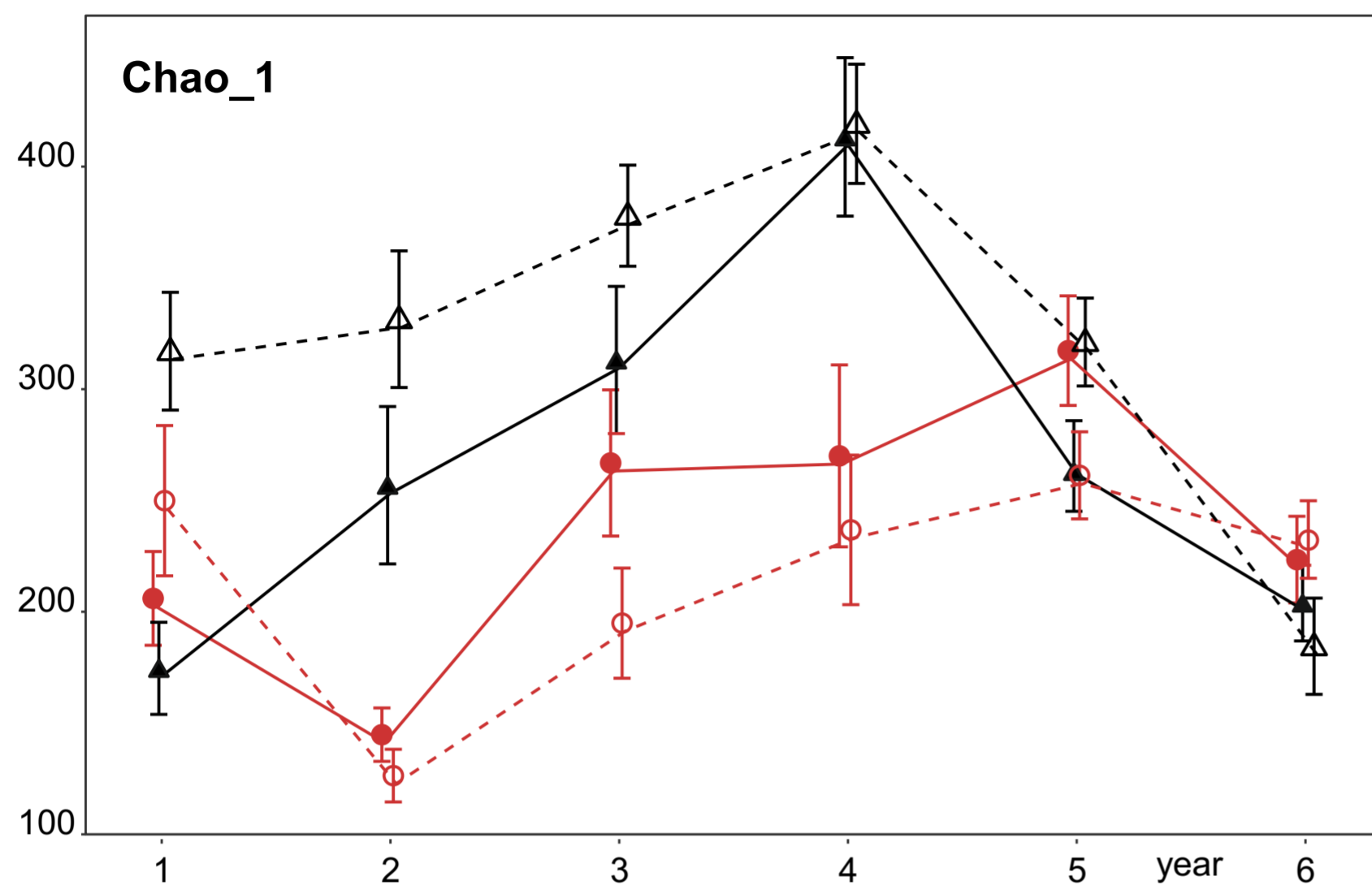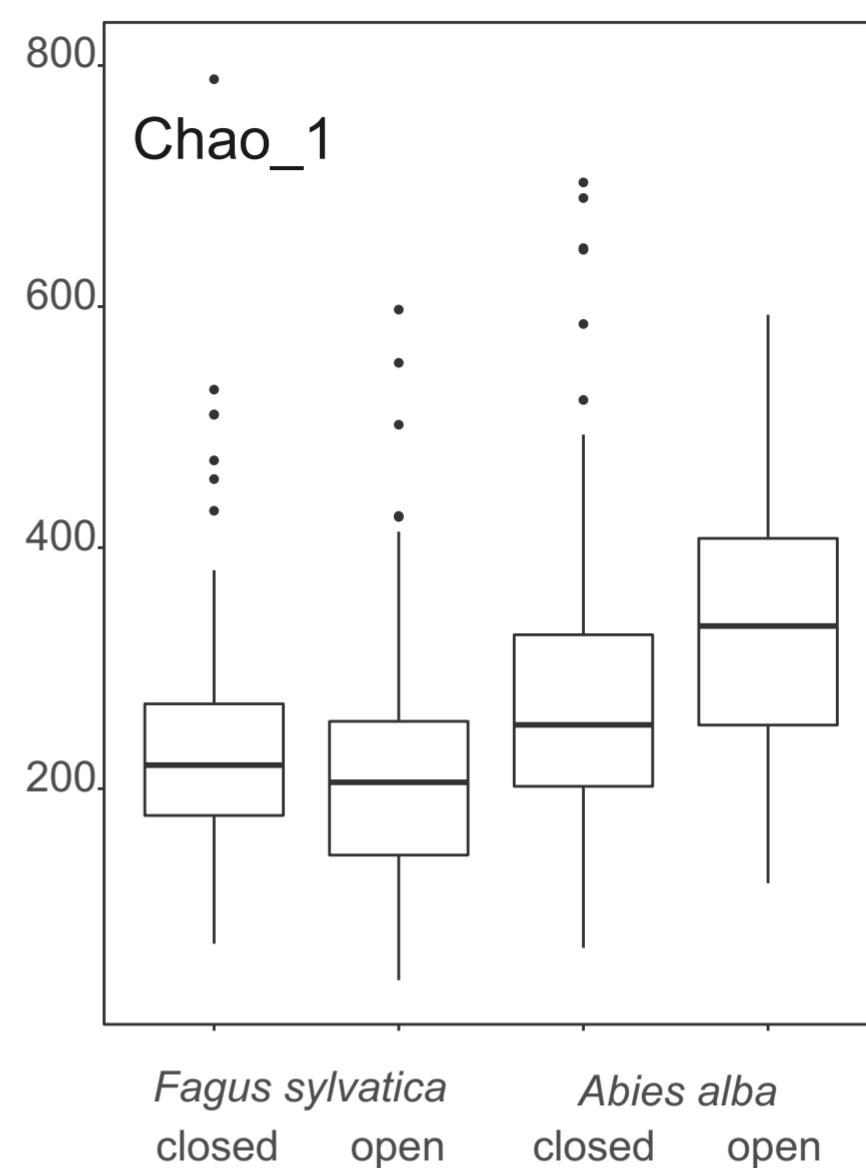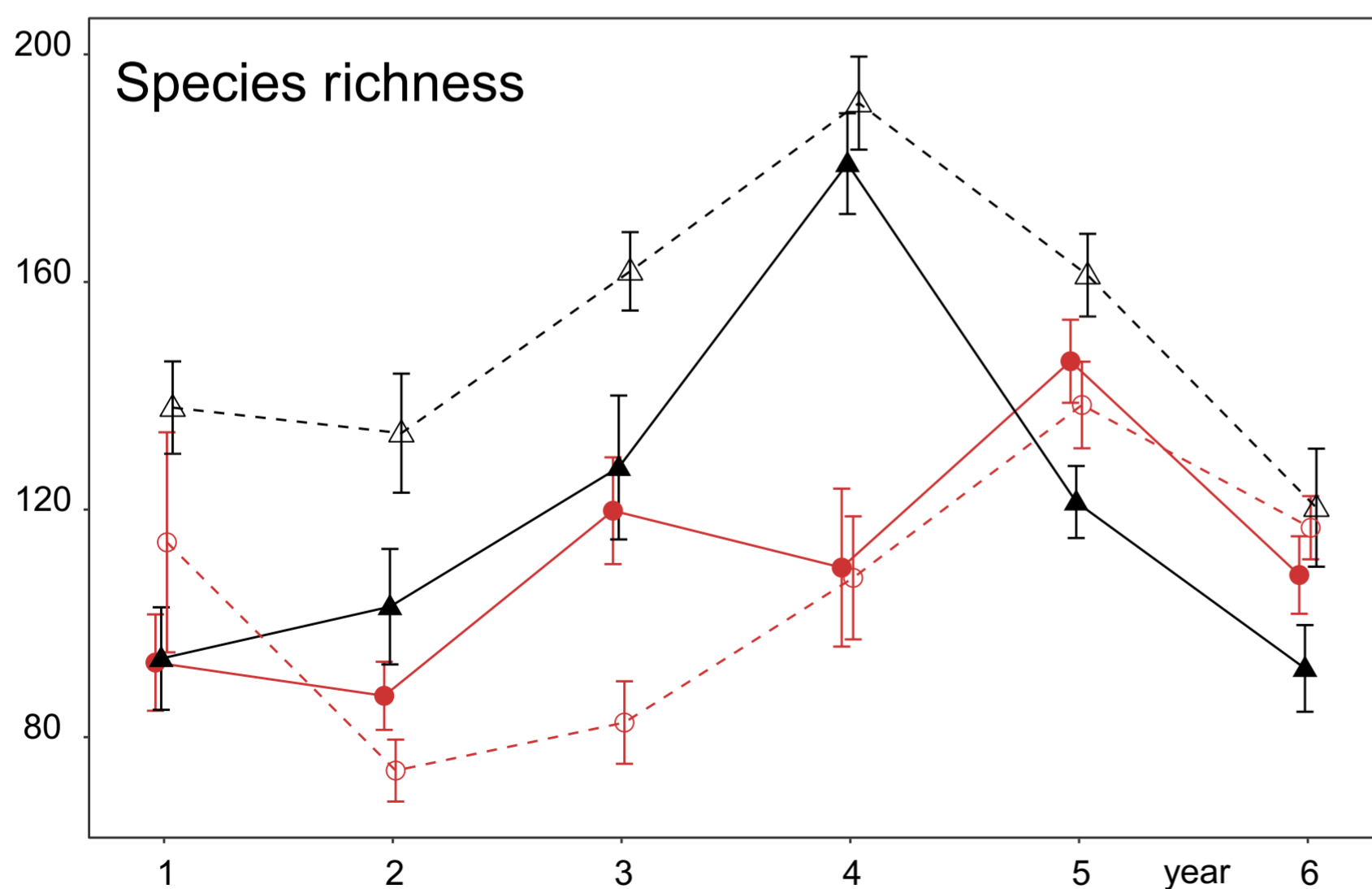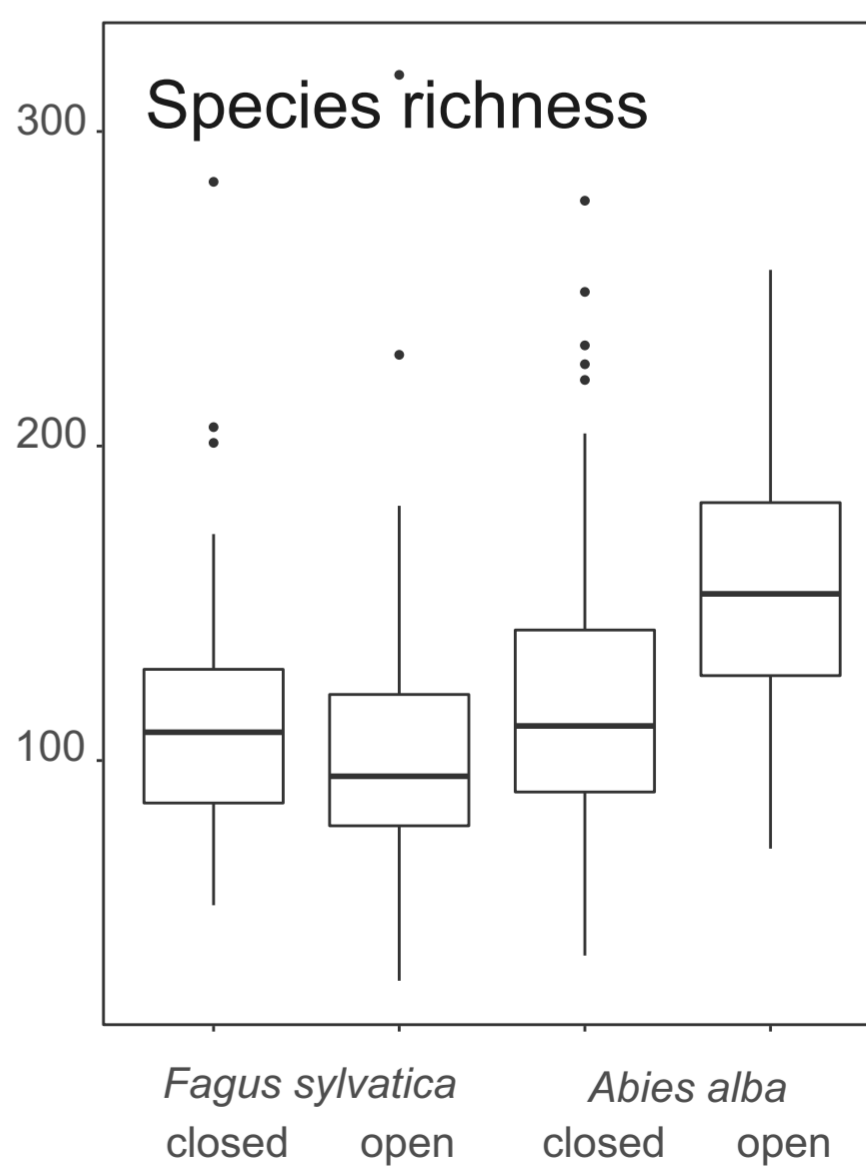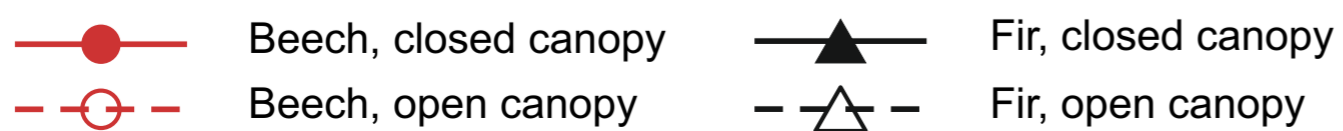

Supplement: Supplementary Figure 4 — Venn diagram representing the results of variation partitioning analyses on Hellinger-transformed OTU abundances. (A) Tree species and canopy cover calculated using all FWD samples, (B) canopy cover, time and deadwood chemism for beech FWD, C: canopy, time and deadwood chemism for fir FWD. [file Data_Sheet_3.PDF]

## A all FWD

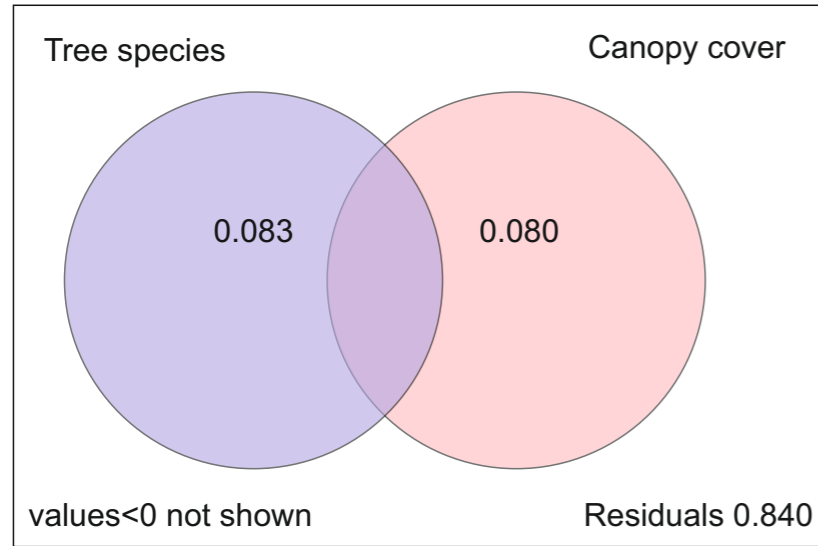

## B *Fagus sylvatica*

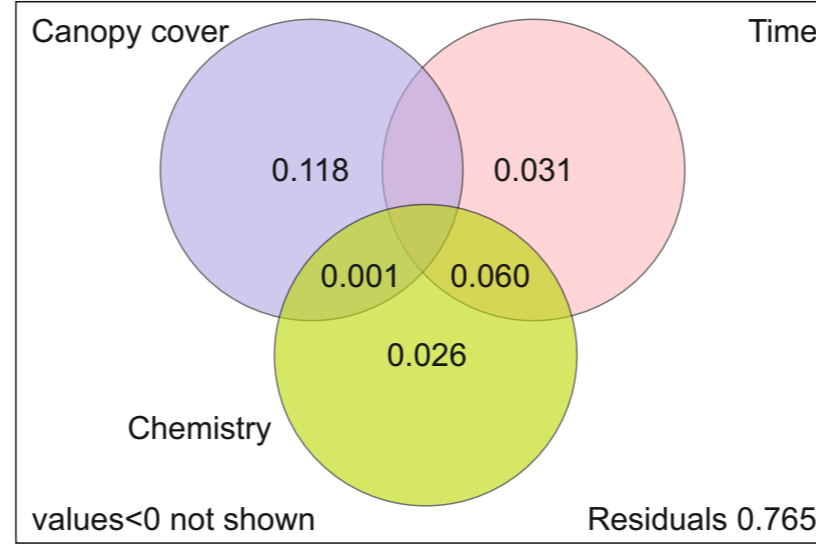

## C *Abies alba*

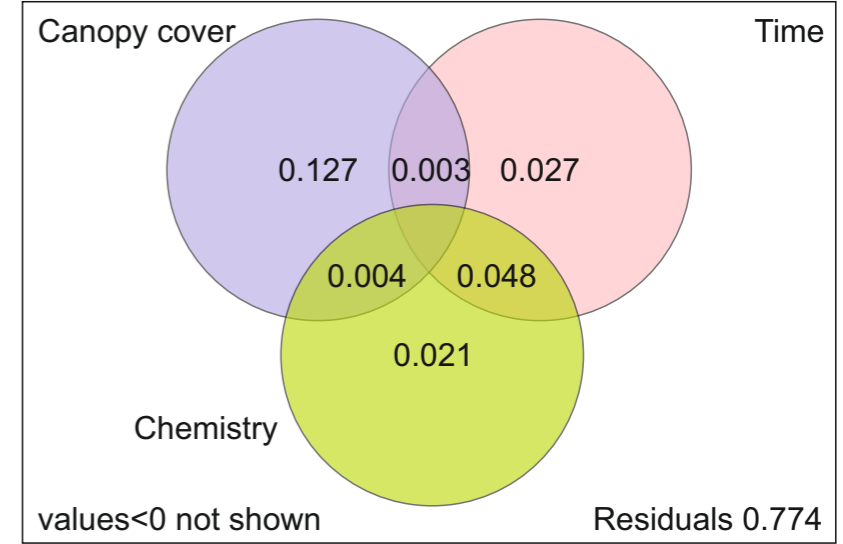

Supplement: Supplementary Figure 5 — Occurrence of fungal taxa during succession on beech and fir fine woody debris (FWD) in a montane forest according to FWD type and microclimatic conditions. Correlations between succession time (in years) of each fungal taxon present on mixed beech and fir FWD (A, P < 0.0001), detected under open and closed canopies in beech (B, P < 0.0001) or fir (C, P < 0.0001) fine woody debris. All taxa with abundances above 0.5% in three or more samples and above 1% in at least one sample were considered. [file Data_Sheet_4.PDF]

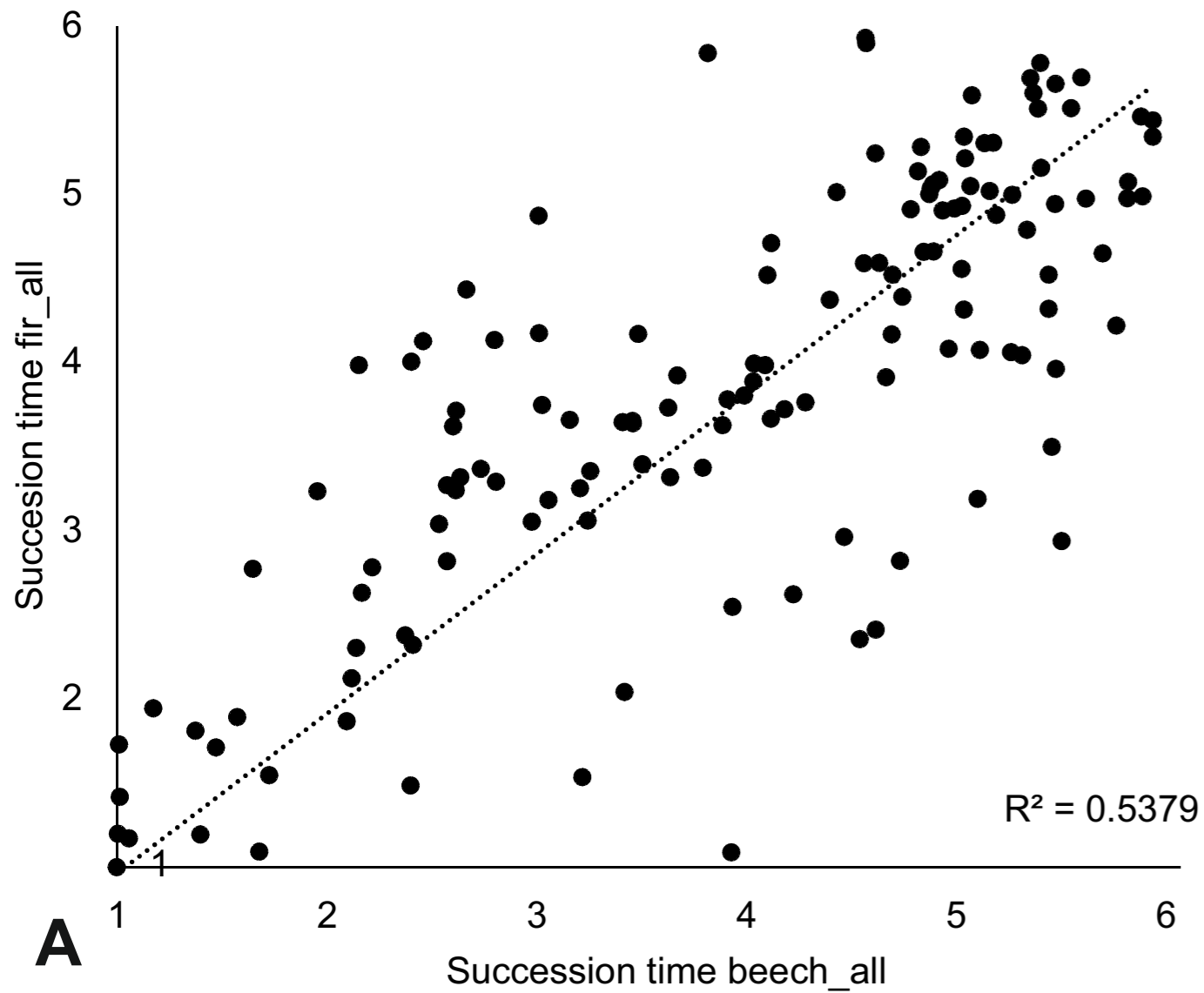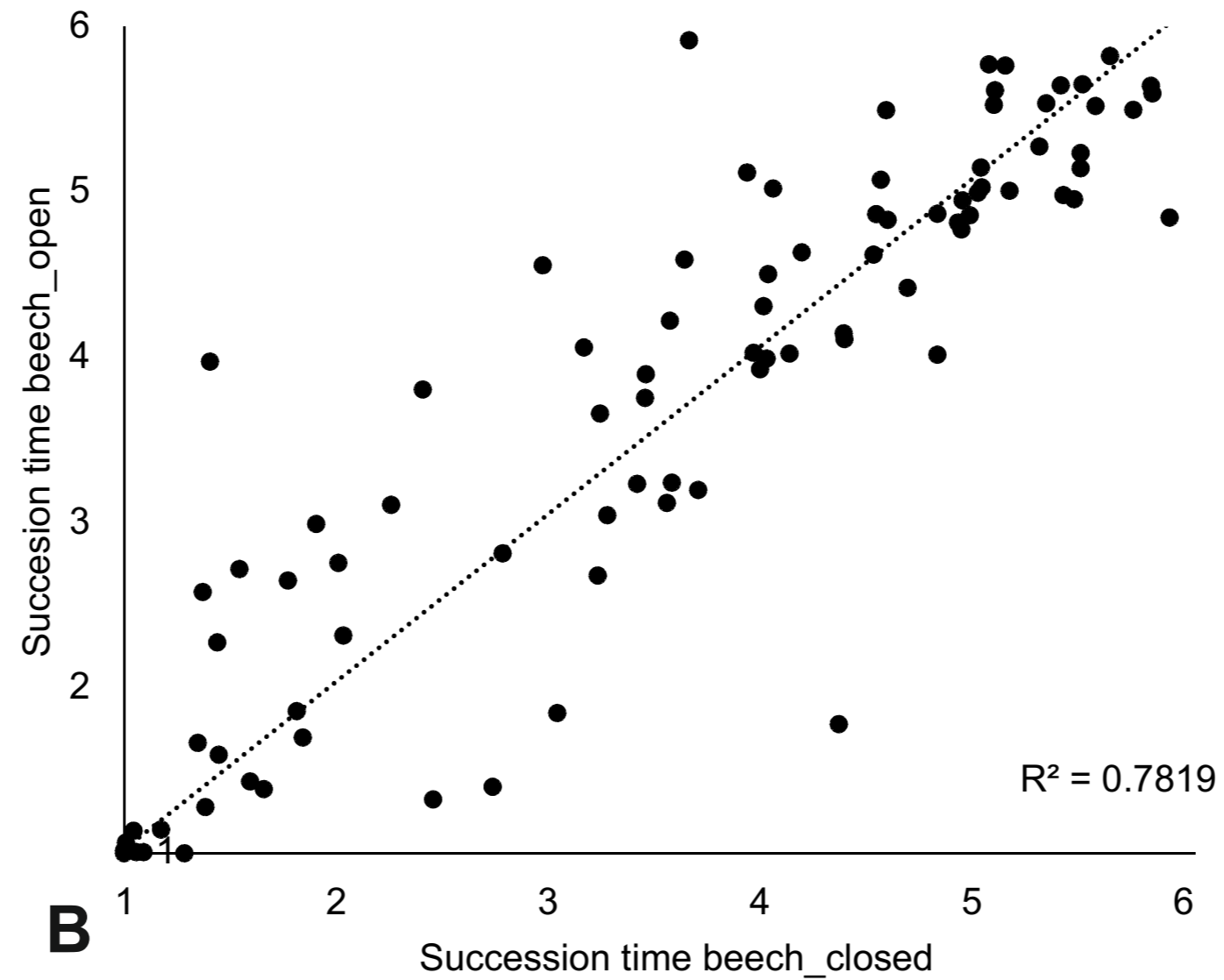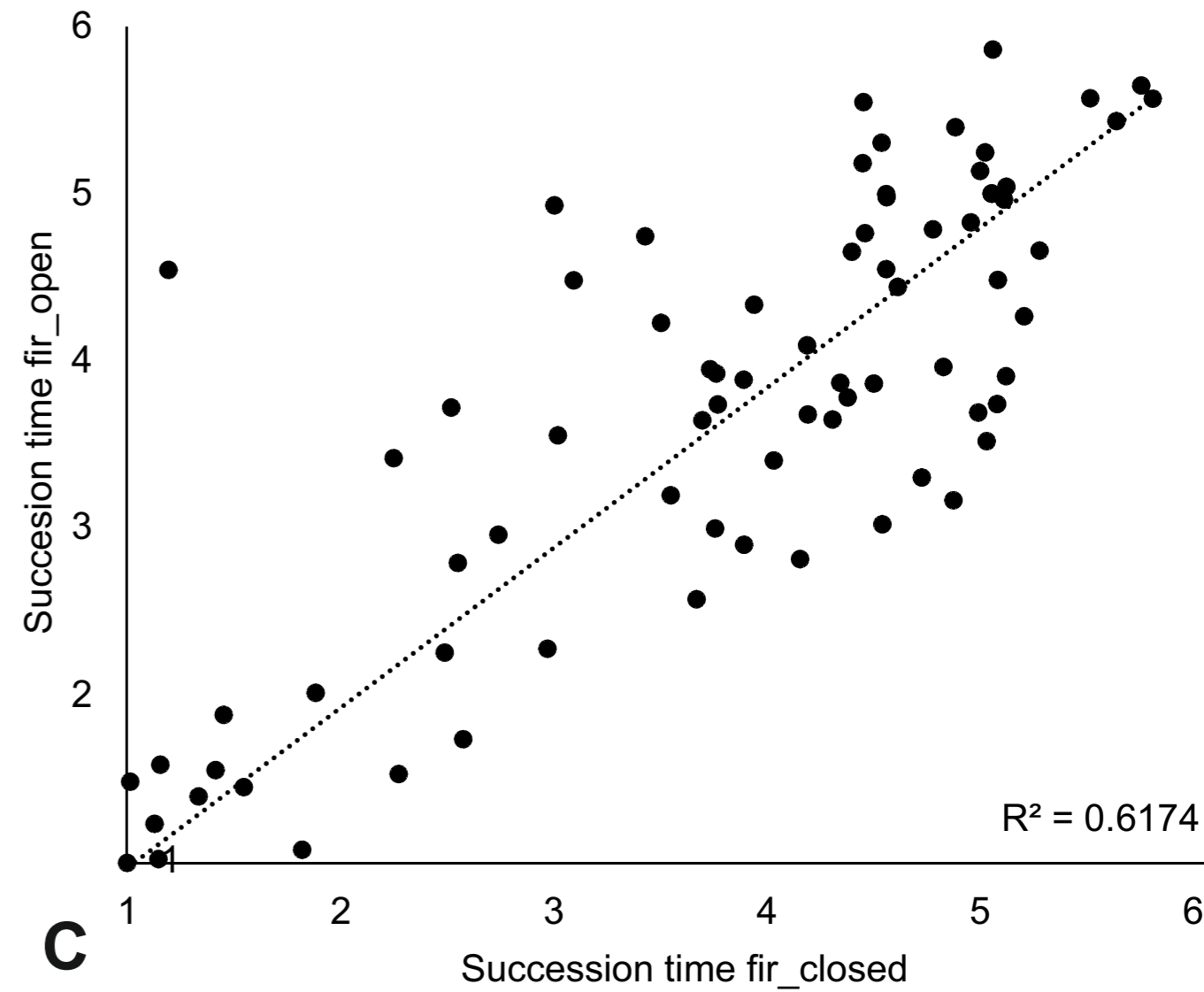

Supplement: Supplementary Figure 6 — Commonly present fungal species and their succession during fine woody debris decomposition. The figure includes species present in at least three samples over 1%, with maximal abundance in a single year over 2% or an average abundance over 0.5%. (A) Fungal species common in beech and fir deadwoods, (B) beech deadwood, both canopy types, (C) fir deadwood, both canopy types. [file Data_Sheet_5.PDF]
